# LncRNA KCNQ1OT1 regulates proliferation and cisplatin resistance in tongue cancer via miR-211-5p mediated Ezrin/Fak/Src signaling

**DOI:** 10.1038/s41419-018-0793-5

**Published:** 2018-07-03

**Authors:** Shanyi Zhang, Hanyu Ma, Daming Zhang, Shule Xie, Weiwei Wang, Qunxing Li, Zhaoyu Lin, Youyuan Wang

**Affiliations:** 1 Guangdong Provincial Key Laboratory of Malignant Tumor Epigenetics and Gene Regulation, Sun Yat-Sen Memorial Hospital, Sun Yat-Sen University, Guangzhou, 510120 China; 2 Department of Neurosurgery, Sun Yat-sen Memorial Hospital, Sun Yat-sen University, Guangzhou, 510120 China; 3 Department of Pathology, The First Affiliated Hospital, Sun Yat-sen University, Guangzhou, 510120 China; 4 Department of Oral & Maxillofacial Surgery, Sun Yat-sen Memorial Hospital, Sun Yat-sen University, Guangzhou, 510120 China; 5 Department of Stomatology, Zibo Center Hospital, Zi Bo, 255000 China

## Abstract

Numerous findings have demonstrated that long noncoding RNA (lncRNA) dysregulation plays a key role in many human neoplasms, including tongue squamous cell carcinoma (TSCC), yet the potential mechanisms of lncRNAs in chemo-resistance remain elusive. Our research showed that the lncRNA KCNQ1OT1 was upregulated in chemo-insensitive TSCC tissues compared with chemo-sensitive TSCC specimens. Meanwhile, high KCNQ1OT1 expression was closely correlated with poor prognosis. Furthermore, KCNQ1OT1 promoted TSCC proliferation and conferred TSCC resistance to cisplatin-induced apoptosis in vitro and in vivo. Using online database analysis, we predicted that the lncRNA KCNQ1OT1 facilitates tumor growth and chemo-resistance by acting as a competing endogenous RNA (ceRNA) to modulate the expression of miR-211-5p. And miR-211-5p upregulation significantly impaired TSCC proliferation and resumed TSCC chemo-sensitivity, which is contrary to the function of lncRNA KCNQ1OT1. Luciferase experiments confirmed that miR-211-5p harbor binding sites for the 3′-UTRof Ezrin mRNA, and Ezrin/Fak/Src signaling was activated in cisplatin-resistant TSCC cells. Finally, miR-211-5p inhibition in sh-KCNQ1OT1-expressing TSCC cells rescued the suppressed cell proliferation and cisplatin resistance induced by KCNQ1OT1 knockdown. In summary, our study has elucidated the role of the oncogenic lncRNA KCNQ1OT1 in TSCC growth and chemo-resistance, which may serve as a new target for TSCC therapy.

## Introduction

Tongue squamous cell carcinoma (TSCC) is one of the most frequently diagnosed malignancies in the oral cavity, and it is associated with a poor prognosis due to its high rate of regional recurrence and lymphoid metastasis^1^. Although aggressive cisplatin chemotherapy is commonly used for tongue cancer treatment and improves overall survival rates, the emergence of chemo-resistance limits its long-term curative effect^2^. The underlying mechanisms resulting in cisplatin resistance in tongue cancer cells remain poorly understood.

Recently, many studies have proven that the dysregulation of noncoding RNAs, including long noncoding RNAs (lncRNAs) and microRNAs (miRNAs), contribute to chemoresistance. lncRNAs are RNA transcripts that are greater than 200 nucleotides but lack protein coding potential, and in multiple tumors, they regulate the expression of genes related to aberrant proliferation and chemoresistance^3,4^. For example, the levels of the lncRNA XIST are significantly upregulated in cisplatin-resistant lung adenocarcinoma cells, and the deletion of XIST contributes to cisplatin-induced cell apoptosis via the let-7i/BAG-1 axis^5^. Similarly, the lncRNA HOXD-AS1 is significantly overexpressed in tongue cancer and promotes proliferation and chemo-resistance by recruiting WDR5^6^. A previous study indicated that the lncRNA MRUL mediated chemo-resistance in gastric cancer cells via regulating ABCB1 expression^7^. Although numerous studies reiterate the importance of lncRNAs in tumor chemoresistance, the molecular mechanisms of TSCC chemo-resistance are not well understood.

miRNAs are evolutionarily conserved small RNAs (20-22 nucleotides long) without protein coding potential. MiRNAs can negatively regulate gene expression post-transcriptionally via binding to complementary sequences on their target mRNAs^8,9^. Aberrantly expressed miRNAs are involved in regulating many cancer-related cellular processes, such as proliferation, migration, apoptosis, stemness, and especially chemoresistance. For instance, miR-205-5p regulates the chemotherapeutic resistance of hepatocellular carcinoma cells by targeting the PTEN/JNK/ANXA3 pathway^10^. MiR-21 may influence cisplatin sensitivity in nasopharyngeal carcinoma cells by targeting PDCD4 and Fas-L^11^. In oral tongue squamous cell cancer, miR-15b may affect cancer-initiating cell phenotypes and cisplatin resistance by targeting TRIM14^12^. Nevertheless, how lncRNAs modulate the miRNAs that regulate chemo-resistance is not well known.

In our study, we screened differentially expressed lncRNAs between three chemo-sensitive tissues and three chemo-insensitive tissues from TSCC patients. We demonstrated that KCNQ1OT1 is most upregulated in chemo-insensitive TSCC samples, and its high expression correlates with poor prognosis in TSCC patients. Furthermore, we identified that KCNQ1OT1 directly modulates the expression of miR-211-5p who harbored binding sites for the 3′-UTRof Ezrin. Both the knockdown of KCNQ1OT1 and the overexpression of miR-211-5p in TSCC cells led to impaired cell proliferation and chemo-resistance. We also found that Ezrin and its downstream Fak/Src signaling activity were inhibited due to KCNQ1OT1 dowregulation. Meanwhile, we found that the impairment of cell proliferation and cisplatin resistance and inhibition of Ezrin/Fak/Src signaling in TSCC cells induced by KCNQ1OT1 knockdown required overexpression of miR-211-5p. Our results confirm for the first time that KCNQ1OT1 promotes cisplatin resistance and cell proliferation by regulating miR-211-5p-mediated Ezrin/Fak/Src signaling, and this lncRNA may be a potential therapeutic target in TSCC patients with cisplatin resistance.

## Results

### lncRNA KCNQ1OT1 is identified as a cisplatin resistant tongue-cancer-related lncRNA, predicts disease prognosis, and mainly locates in cytoplasm

**Fig. 1:**
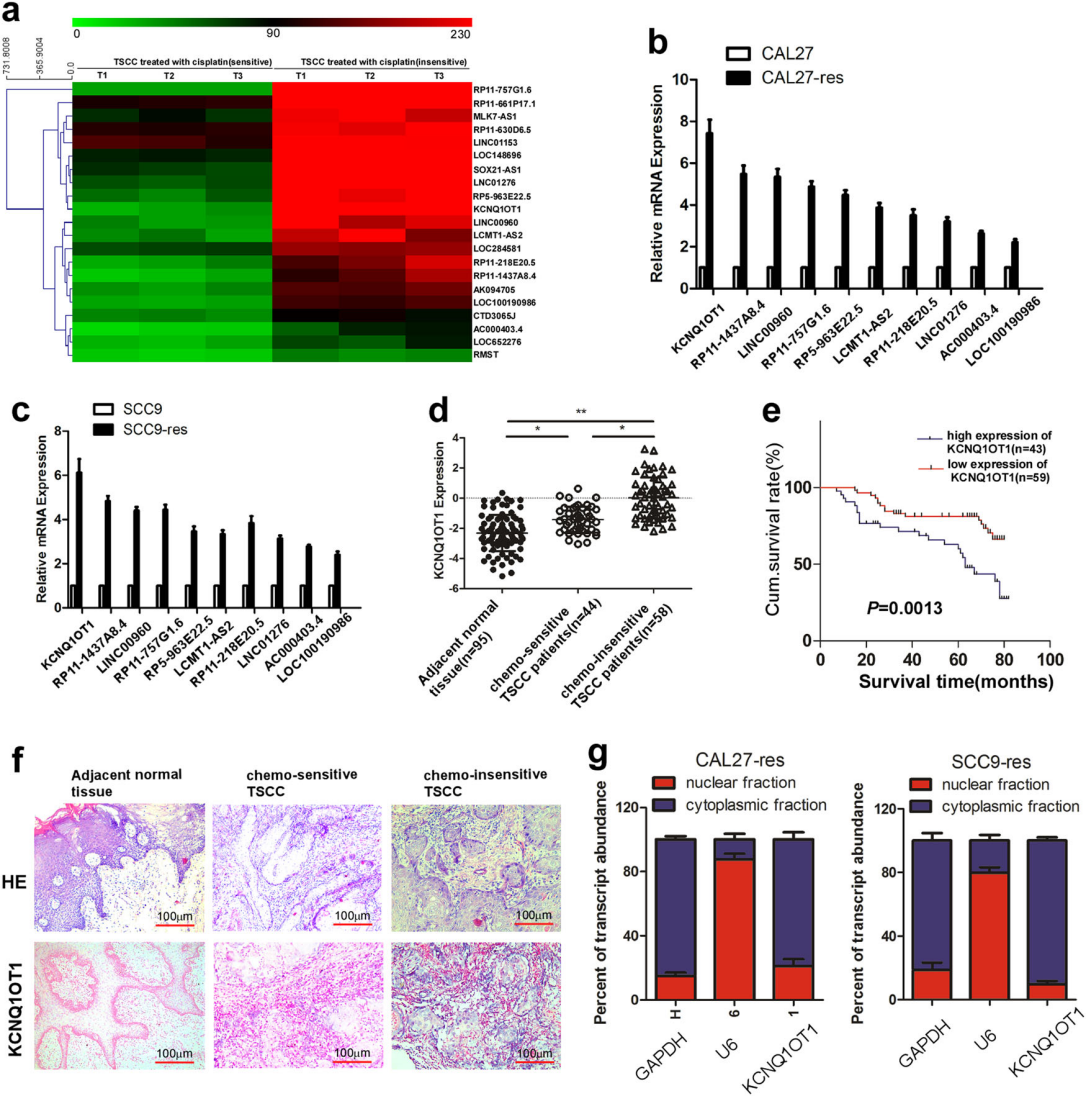
KCNQ1OT1 is identified as a cisplatin resistant tongue-cancer-related lncRNA, predicts disease prognosis and mainly locates in cytoplasm. **a**The differentially expressed lncRNAs between three chemo-insensitive tissues and three chemo-sensitive tissues were detected using a microarray. **b,c** The results from microarray analysis were validated in cisplatin-resistant tongue cancer cells and their parental cells by RT-qPCR. **d** The expression of KCNQ1OT1 in adjacent normal tissues, chemo-sensitive samples and chemo-insensitive TSCC tissues was examined by RT-qPCR. **e** The progression-free survival rates of 102 TSCC patients were compared in the KCNQ1OT1-low and KCNQ1OT1-high groups. **f** Representative images of in situ hybridization for KCNQ1OT1in paraffin-embedded TSCC tissues and adjacent normal tissues (scale bar: 100 μM). **g** The proportion of KCNQ1OT1 in CAL27-res and SCC9-res cells was detected by RT-qPCR

Using lncRNA microarray analysis, we detected aberrantly expressed lncRNAs between three chemo-sensitive tissues and three chemo-insensitive tissues from TSCC patients (Fig. 1a). Using RT-qPCR, we further validated the expression levels of differential lncRNAs in cisplatin-resistant TSCC cells compared with their parental cells, which we had previously constructed^13^. Then we focused on the top ten upregulated lncRNAs in chemo-insensitive samples, and interestingly, lncRNA KCNQ1OT1 was the most upregulated lncRNA in both chemo-insensitive TSCC samples and cisplatin-resistant TSCC cells (Fig. 1b, c).

Next we examined KCNQ1OT1 expression levels in 95 adjacent normal tissues and 102 TSCC tissues that were divided into either the chemo-insensitive group (*N* = 58) and chemo-sensitive group (*N* = 44). As shown in Fig. 1d, the KCNQ1OT1 level was increased significantly in TSCC specimens compared with adjacent normal tissues. Moreover, the expression levels of KCNQ1OT1 were higher in TSCC tissues obtained from chemo-insensitive TSCC patients than that in chemo-sensitive TSCC samples (*p* < 0.01). We also found that the expression of KCNQ1OT1 was correlated with Gleason score, T stage, and lymph node status (Table 1). In addition, Kaplan-Meier survival analysis indicated that increased KCNQ1OT1 expression in TSCC tissues was significantly associated with a lower rate of overall survival (*p* < 0.01) (Fig. 1e). Finally, using in situ hybridization (ISH) and RT-qPCR arrays, we found that KCNQ1OT1 was mainly expressed in the cytoplasm (Fig. 1f, g). These results indicated that the lncRNA KCNQ1OT1 may be involved in the occurrence and development of chemo-resistance, and it may serve as a potential marker to predict chemo-responsiveness and prognosis in TSCC patients.

**Table 1:** Correlation among clinicopathologic status and the expression of KCNQ1OT1 in TSCC patients

Characteristic	KCNQ1OT1(%)		*P*
	No. of high expression	No. of low expression	
Sex			0.557
Male	23(39.7)	35(60.3)	
Female	20(45.5)	24(54.5)	
Age			0.994
<50	16(42.1)	22(57.9)	
≥50	27(42.2)	37(57.8)	
Node metastasis			0.130
N0	19(35.2)	35(64.8)	
N+	24(50.0)	24(50.0)	
Clinical stage			0.807
III	20(43.5)	26(56.5)	
IV	23(41.1)	33(58.9)	
Status			0.002
Survival	18(29.5)	43(70.5)	
Death	25(61.0)	16(39.0)	
Cisplatin			0.001
Sensitive	10(22.7)	34(77.3)	
Non-sensitive	33(56.9)	25(43.1)	

### Knockdown of KCNQ1OT1 inhibits TSCC cell proliferation and chemoresistance

**Fig. 2:**
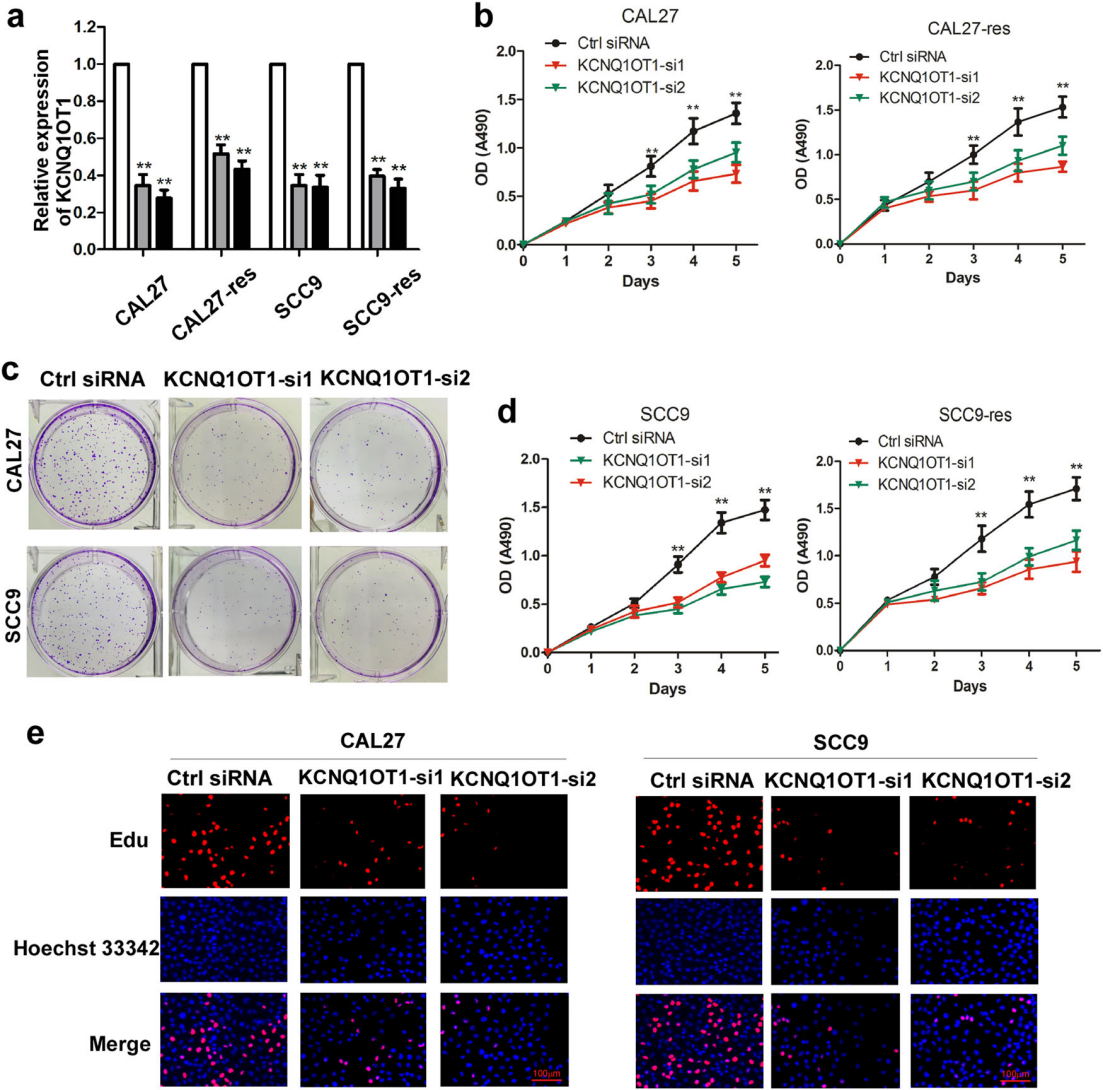
Knockdown of KCNQ1OT1 inhibits tongue cancer cell proliferation. **a** Efficiency of KCNQ1OT1 knockdown in CAL27-res/CAL27 and SCC9-res/SCC9 cells by two siRNAs was verified by RT-qPCR. **b** Influence of KCNQ1OT1 knockdown on cell viability of CAL27-res and CAL27 cells was measured by the MTS assay. **c** Effect of KCNQ1OT1 knockdown on flat colony formation was measured in SCC9 and CAL27 cells. **d** Effect of KCNQ1OT1 knockdown on cell viability of SCC9-res and SCC9 cells was measured by the MTS assay. **e** CAL27 and SCC9 cells were transfected with KCNQ1OT1 siRNAs or control siRNA and the cell viability were examined by EDU assay

To further elucidate the role of KCNQ1OT1 in TSCC progression, we downregulated KCNQ1OT1 expression in TSCC cells using two specific siRNAs targeting KCNQ1OT1. KCNQ1OT1 was markedly inhibited in CAL27, SCC9, CAL27-res, and SCC9-res cells after transfecting siRNAs targeting KCNQ1OT1 (Fig. 2a). The MTS assay showed that KCNQ1OT1 downregulation significantly inhibited cell growth in cisplatin-resistant cells and their parental cells (Fig. 2b, d). Consistent with this data, compared with the control group, the group with KCNQ1OT1 knocked down had fewer and smaller colony formations (Fig. 2c, Supplementary Fig. 1a). In addition, using EdU assays, we observed a decrease in cell division after KCNQ1OT1 was deleted (Fig. 2e, Supplementary Fig. 1b).

**Fig. 3:**
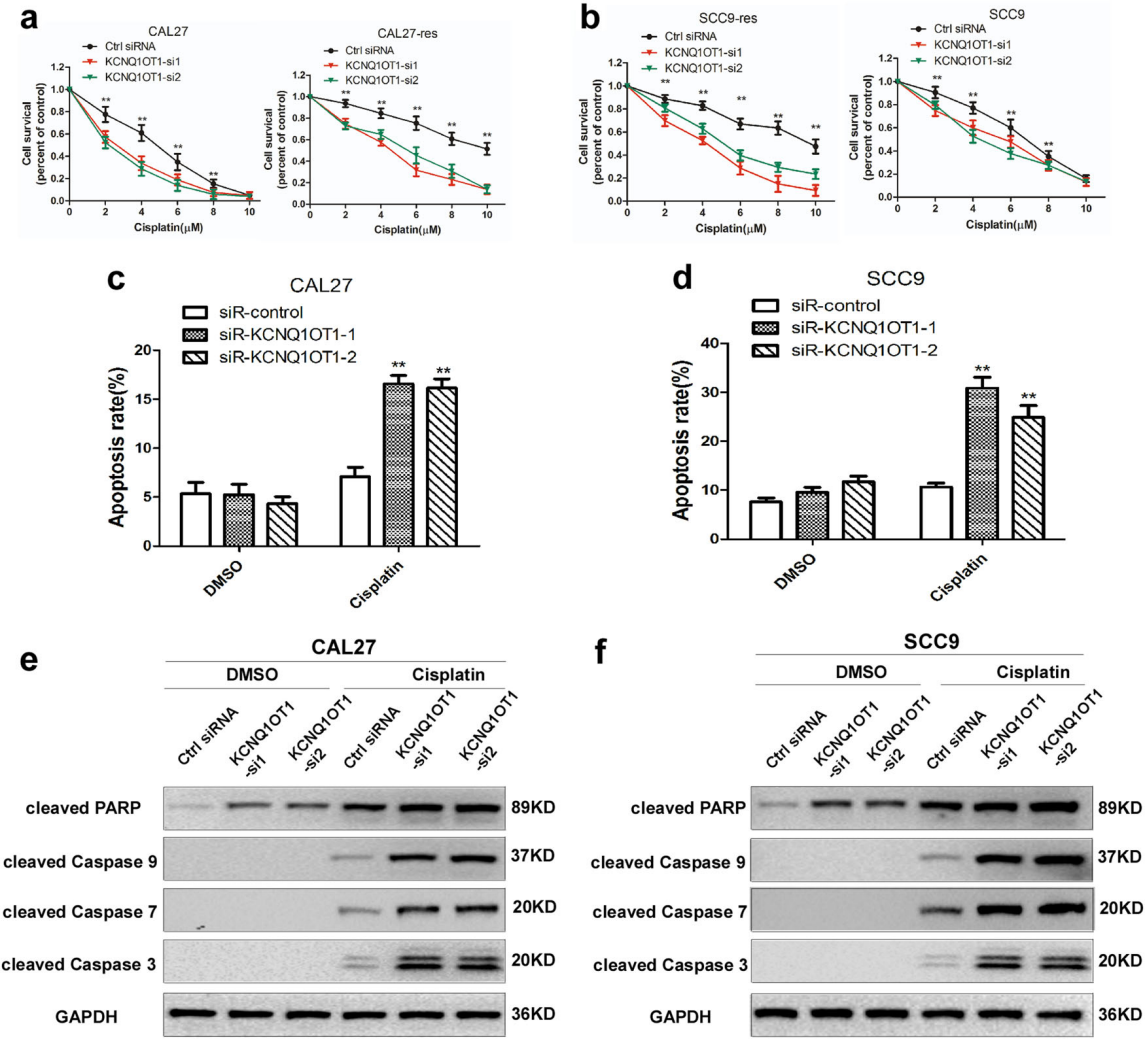
Downregulation of KCNQ1OT1 represses chemo-resistance of tongue cancer cells. **a,b** CAL27-res/CAL27 cells (**a**) and SCC9-res/SCC9 cells (**b**) transfected with KCNQ1OT1 siRNAs or control siRNA were treated with different concentration of cisplatin for 24 h and the cell survival rate was analyzed by MTS assay. **c,d** The CAL27 (**c**) and SCC9 (**d**) cells transfected with control or KCNQ1OT1 siRNAs were treated with 4 μM cisplatin (for CAL27 cells) or 8 μM cisplatin(for SCC9 cells) for 24 h. The percentage of apoptotic cells was analyzed by flow cytometer. **e,f** KCNQ1OT1-depleted CAL27 (**e**) and SCC9 (**f**) cells were treated with either DMSO or 4 μM cisplatin(for CAL27 cells) and 8 μM cisplatin(for SCC9 cells) for 24 h, and the expression of cleaved PARP and cleaved caspase-3, -7, and -9 were detected by Western blot analysis

Next, we detected whether KCNQ1OT1 deletion influenced the resistance to cisplatin in TSCC cells in a dose-dependent manner. Interestingly, compared with the cisplatin sensitivity of control cells, KCNQ1OT1 inhibition sensitized CAL27, SCC9, CAL27-res, and SCC9-res cells to cisplatin treatment (Fig. 3a, b), indicating that KCNQ1OT1 upregulation contributes to chemo-resistance in TSCC. Flow cytometric analysis showed that the inhibition of KCNQ1OT1 levels alone induced mild levels of apoptosis in CAL27 and SCC9 cells, whereas the apoptosis rate increased significantly after cisplatin treatment in TSCC cells (Fig. 3c, d, Supplementary Figs 1c and 1d). Furthermore, we detected markers of apoptosis, such as cleaved PARP and caspase proteins in CAL27 and SCC9 cells. After cisplatin treatment, cells transfected with siRNAs targeting KCNQ1OT1 had increased expression levels of cleaved PARP and cleaved caspase-3, -7, and -9 (Fig. 3e, f). In order to reduce the off-target effect of siRNAs, we also inhibited KCNQ1OT1 expression using a “CRISPR Interference” (CRISPRi) method. As showed in Supplementary Fig. 2a, sgRNA5 and sgRNA6 have a similar inhibitory effect and sgRNA6 was used for next biological experiments. KCNQ1OT1 downregulation in CAL27-res and SCC9-res cells enhanced the chemo-sensitivity to cisplatin (Supplementary Fig. 2e, f), but inhibited TSCC growth (Supplementary Fig. 2b–d).

Furthermore, we ectopically expressed KCNQ1OT1 expression using a newly-developed technologies “CRISPR Activation” (CRISPRa) for its higher stability and lower off-target effect^14,15^. We used designed sgRNAs (sgRNA1~3) targeting the dCas9-VP64 protein to the promoter regions of KCNQ1OT1. CAL27 and SCC9 cells tranfected with The expression of these sgRNAs induced various significant increases in The expression levels of KCNQ1OT1 significantly increased in CAL27 and SCC9 cells tranfected with plasmids containing these sgRNAs and the sgRNA-3 transfection induced the strongest activation of KCNQ1OT1 expression (Supplementary Fig. 3a). Meawhile, the sgRNA-3 activation on KCNQ1OT1 promoter significantly promoted cell growth in CAL27 and SCC9l cells detected by MTS assays, colony formation and EdU assays (Supplementary Fig. 3b-d). In addition, KCNQ1OT1 overexpression in CAL27 and SCC9 cells remarkably restored their sensitivities to cisplatin. The numbers of apoptotic cells were decreased, and more tongue cancer cell were suvived under cisplatin pressure due to the KCNQ1OT1 upregulation (Supplementary Fig. 3e,f). Collectively, these data indicated that long noncoding RNA KCNQ1OT1 could promote cell proliferation and enhance the chemo-resistance in TSCC cells.

### KCNQ1OT1 binds to miR-211-5p and represses their expression

**Fig. 4:**
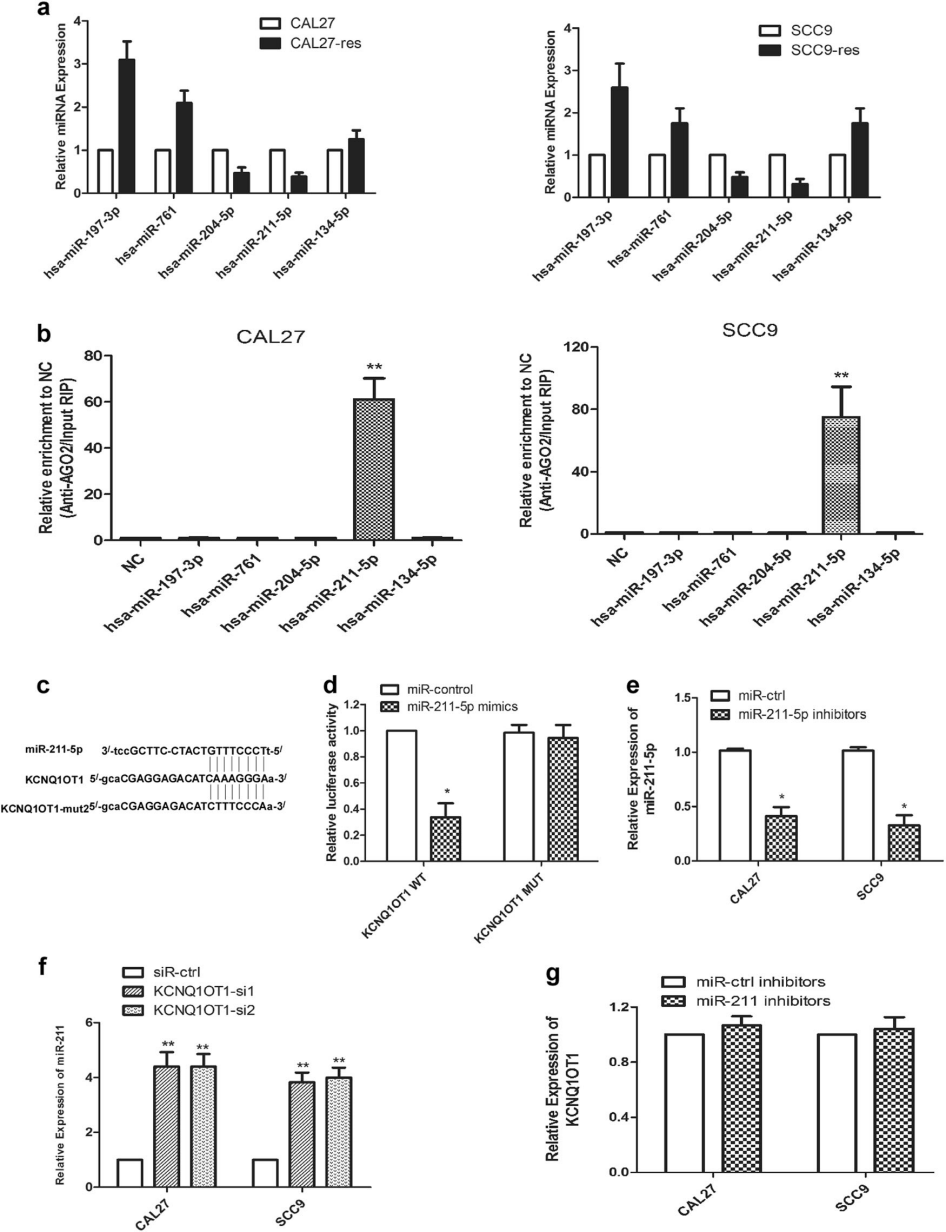
KCNQ1OT1 binds to miR-211-5p and represses their expression. **a** Differentially expressed miRNAs were validated in CAL27-res/CAL27 cells and SCC9-res/SCC9 cells by RT-qPCR. **b** Anti-AGO2 RIP was performed in CAl27 and SCC9 cells transiently overexpressing miRNA mimics, followed by RT-qPCR to detect lncRNA KCNQ1OT1 associated with AGO2. **c** Schematic illustration of the predicted binding sites between KCNQ1OT1 and miR-211-5p and mutation of potential miR-211-5p binding sequence in KCNQ1OT1. **d** Luciferase assays in 293T cells transfected with wild-type or mutant KCNQ1OT1 and miR-211-5p. **e** The expression of miR-211-5p was inhibited in CAL27 and SCC9 transfected with miR-211-5p inhibitors or miR-control by RT-qPCR. **f** The expression of miR-211-5p was upregulated in CAL27 and SCC9 transfected with KCNQ1OT1 siRNAs or control siRNA by RT-qPCR. **g** The expression of KCNQ1OT1 was inhibited in CAL27 and SCC9 transfected with miR-211-5p inhibitors or miR-control by RT-qPCR

An increasing amount of evidence shows that lncRNAs may act as miRNA sponges to regulate the binding of endogenous miRNAs to their target mRNAs and to inhibit the expression of these target mRNAs^16,17^. Using bioinformatic tools starBase v2.0 (http://starbase.sysu.edu.cn/), we predicted 89 miRNAs with potential to interact with KCNQ1OT1(data shown in the S2-table). Furthermore, a miRNA microarray was used to screen differentially expressed miRNAs with potential to interact with KCNQ1OT1 in the paired sh-KCNQ1OT1 and sh-NC CAL27 cells. As shown in Supplementary Fig. 4a and b, we found that there were five upregulated miRNAs including hsa-miR-197-3p, hsa-miR-761, hsa-miR-204-5p, hsa-miR-211-5p, hsa-miR-134-5p, who could also be found in starBase. Then we performed RT-qPCR assay to study the expression level of five miRNAs in cisplatin resistant cells and their parental cells. Downregulation of miR-204-5p and miR-211-5p were found in CAL27-res and SCC9-res cells (Fig. 4a). The microRNAs can bind their target genes and repress translational levels in an AGO2-dependent manner. To determine whether lncRNA KCNQ1OT1 was regulated by the upregulated miRNAs in an AGO2-dependent manner, we conducted anti-AGO2 RIP in CAL27 and SCC9 cells transiently overexpressing these miRNAs. Endogenous lncRNA KCNQ1OT1 pull-down by AGO2 was specifically enriched in miR-211-5p overexpressing cells (Fig. 4b). Further bioinformatic analyses revealed the miRNA response elements (MREs) for miR-211-5p in the KCNQ1OT1 sequence (Fig. 4c). Next, we cloned wild-type KCNQ1OT1 luciferase plasmids containing potential miR-211-5p binding sites or mutants for each site. These plasmids were co-transfected with miR-211-5p into HEK293T cells, and then, luciferase assays were performed to investigate whether miR-211-5p bind to KCNQ1OT1. As shown in Fig. 4d, co-transfection of miR-211-5p with wild-type KCNQ1OT1 substantially inhibited luciferase activity, but they did not affect the luciferase activity of the KCNQ1OT1 mutants. Then we found that miR-211-5p expression was upregulated when we knockdown KCNQ1OT1 (Fig. 4f). The results suggested that the miR-211-5p binding sites within KCNQ1OT1 are functional. Furthermore, we investigated whether inhibition of miR-211-5p in CAL27 and SCC9 cells could not cause any change in expression level of KCNQ1OT1 (Fig. 4e, g). In summary, our data supported that miR-211-5p as inhibitory targets of KCNQ1OT1 in TSCC.

### miR-211-5p suppress TSCC proliferation and chemo-resistance by targeting Ezrin/Fak/Src signaling

**Fig. 5:**
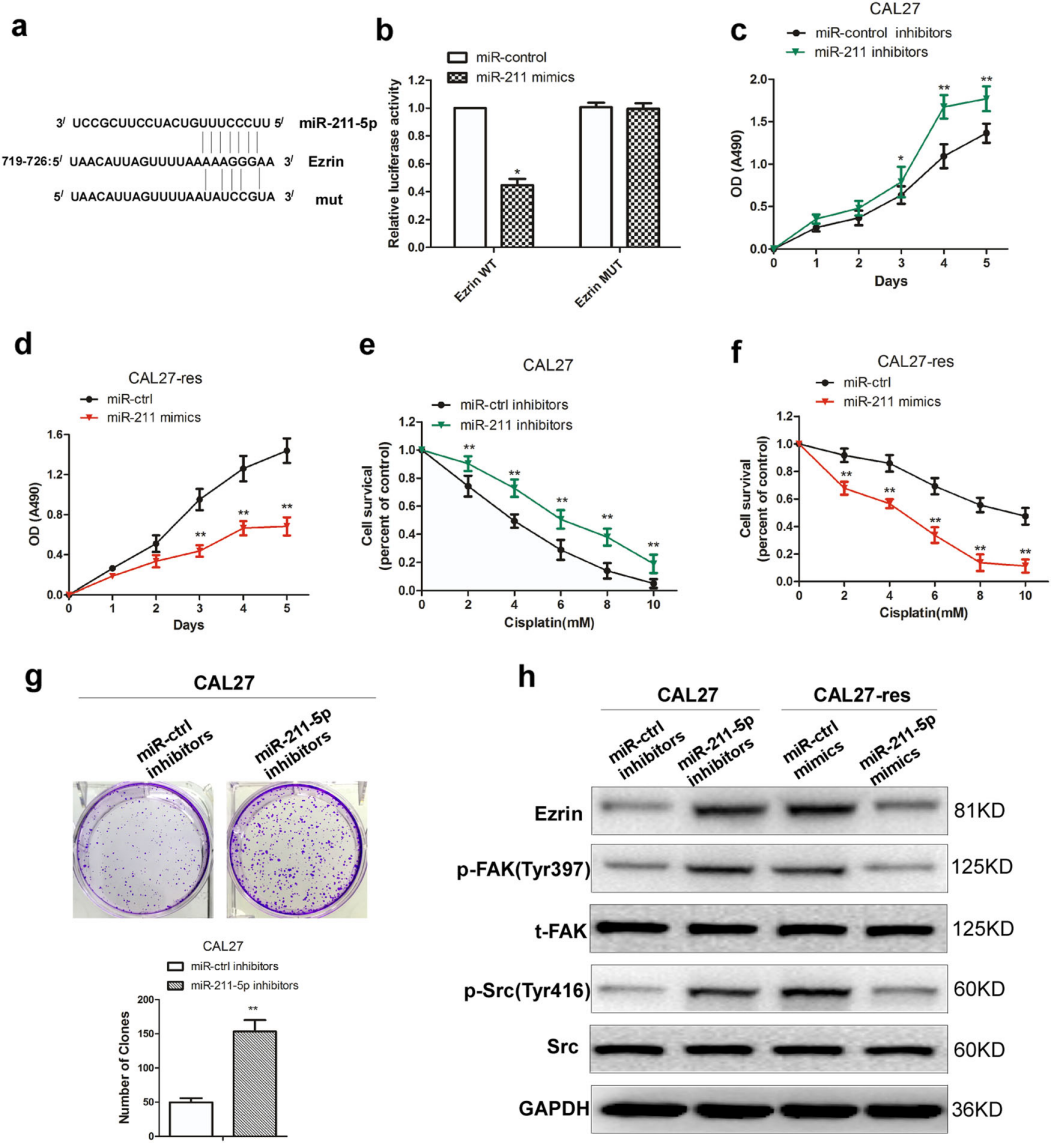
miR-211-5p suppress TSCC proliferation and chemo-resistance by targeting Ezrin/Fak/Src signaling. **a** Target sequence of miR-211-5p in Ezrin 3′-UTR predicted by TargetScan and miRanda as well as the mutated sequence. **b** Luciferase assays in 293 T cells transfected with wild-type or mutant Ezrin and miR-211-5p mimics. **c** Cell proliferation was detected by MTS assays in CAL27 cells transfected with or without miR-211-5p inhibitors. **d** Cell proliferation was detected by MTS assays in CAL27-res cells transfected with or without miR-211-5p mimics. **e** Cell survival capacity was examined by MTS assays under cisplatin pressure in CAL27 cells transfected with or without miR-211-5p inhibitors. **f** Cell survival rate was examined by MTS assays under cisplatin pressure in CAL27-res cells transfected with or without miR-211-5p mimics. **g** Clones formation was detected in CAl27 cells transfected with miR-211-5p inhibitors. **h** The expression of Ezrin, p-Fak, total Fak, p-Src, and total Src were detected in miR-211-5p-depleted CAL27 cells and miR-211-5p overexpressed CAL27-res cells by Western blot analysis

To further investigate the potential role of miR-211-5p on TSCC proliferation and chemo-resistance. miR-211-5p mimics were transfected into CAL27 and SCC9 cells, and miRNA inhibitors against miR-211-5p were also transfected into cisplatin-resistant TSCC cells. Compared with cells in the control group, the CAL27-res and SCC9-res cells overexpressing miR-211-5p had significantly decreased cell proliferation (Fig. 5d, Supplementary Fig. 5b) and chemo-resistance (Fig. 5f, Supplementary Fig. 5d), while miR-211-5p inhibition in CAL27 and SCC9 cells promoted cell proliferation (Fig. 5c, g, Supplementary Fig. 5a and f) and chemo-resistance (Fig. 5e, Supplementary Fig. 5c). These data indicated that miR-211-5p contributed to tumor suppression by reducing cell growth and chemo-resistance in TSCC, which is contrary to the role of KCNQ1OT1 lncRNA.

We next used several bioinformatics databases (Targetscan and miRanda) to predict potential target genes of miR-211-5p. It is obvious that Ezrin (EZR) is a common target gene of miR-211-5p (Fig. 5a). To verify whether Ezrin was a functional target of miR-211-5p, based on the potential miR-211-5p binding sites on the EZR 3′-UTR, we cloned luciferase reporter plasmids containing wild-type and mutated 3′-UTR EZR binding sites. Dual luciferase reporter assays showed that miR-211-5p overexpression significantly reduced the luciferase activity driven by the wild-type EZR 3′-UTR, but did not influence luciferase activities in the presence of mutant EZR 3′-UTRs (Fig. 5b). The dysregulation of Ezrin has been well documented in many types of human malignancies, and previous studies have shown that the overexpression and activation of Ezrin alter cell shape, adhesion, motility and apoptosis and correlate with the invasion and metastasis of many human cancers^18,19^. Next, we explored whether miR-211-5p negatively regulate Ezrin. As shown in Fig. 5h and Supplementary Fig. 5e, the protein levels of Ezrin were higher in CAL27-res and SCC9-res cells, but Ezrin was decreased in cisplatin resistant TSCC cells transfected with miR-211-5p mimics. Meanwhile, the protein levels of Ezrin were increased when we inhibited miR-211-5p expression in CAL27 and SCC9 cells (Fig. 5h and Supplementary Fig. 5e). Since Ezrin is an important component of the Fak/Src signaling pathway, we next sought to determine whether miR-211-5p affect the activity of Fak/Src signaling. Our results indicated that Fak and Src phosphorylation were reduced in CAL27-res and SCC9-res cells with miR-211-5p overexpression, yet the phosphorylation levels of Fak and Src were enhanced by miR-211-5p inhibition in CAL27 and SCC9 cells (Fig. 5h and Supplementary Fig. 5e). According to the above findings, miR-211-5p act as tumor suppressors by targeting Ezrin/Fak/Src signaling in TSCC.

### KCNQ1OT1 promotes TSCC progression via miR-211-5p-mediated Ezrin/Fak/Src signaling

**Fig. 6:**
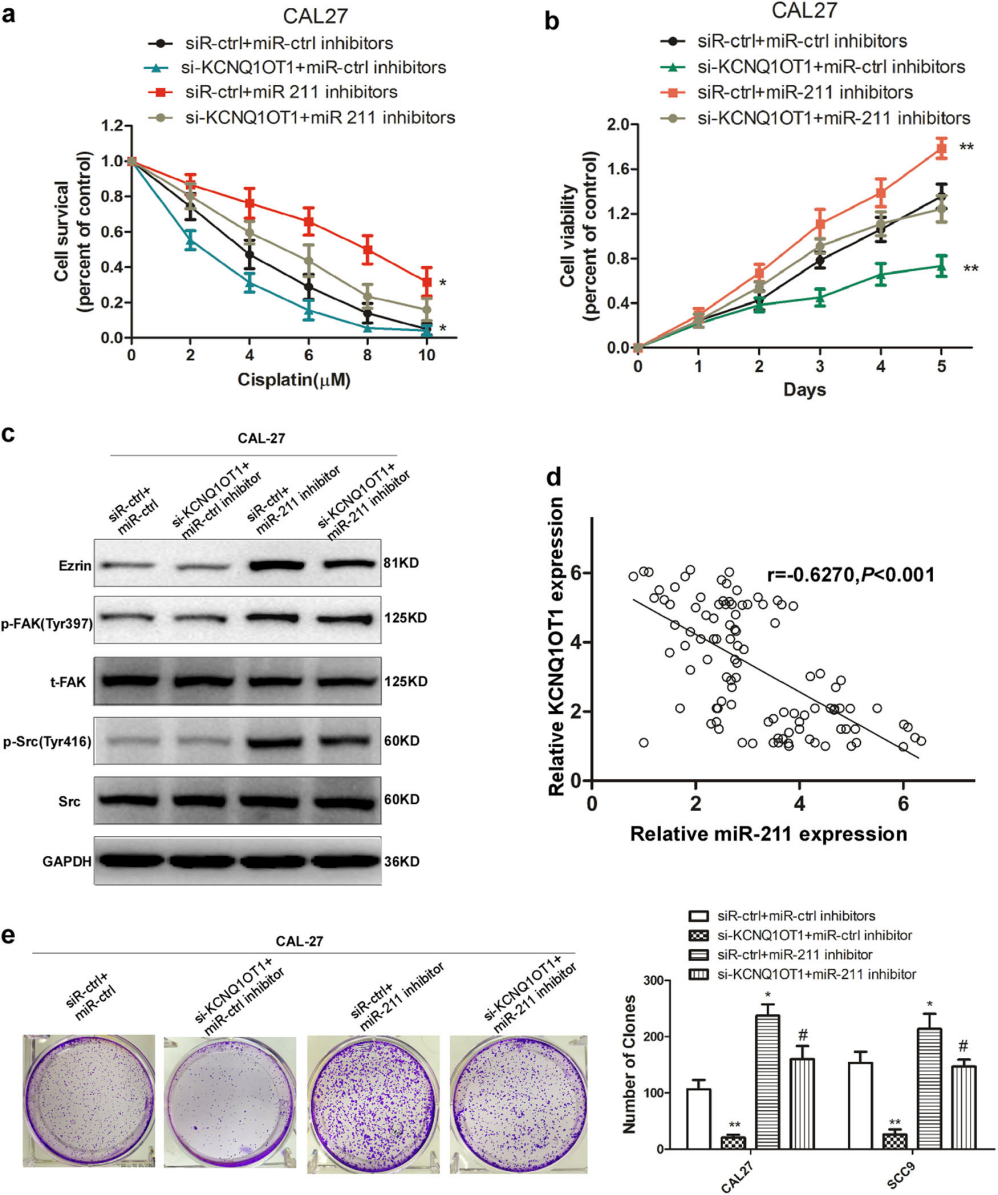
KCNQ1OT1 promotes TSCC progression via miR-211-5p-mediated Ezrin/Fak/Src signaling. **a** Cell survival rate of CAL27 cells transfected with siR-KCNQ1OT1 or siR-control simultaneously with miR-211-5p inhibitors or miR-control inhibitors under cisplatin pressure were examined with MTS assays. **b** Cell proliferation ability of CAL27 cells transfected with siR-KCNQ1OT1 or siR-control simultaneously with miR-211-5p inhibitors or miR-control inhibitors under cisplatin pressure were examined with MTS assays. **c** The expression of Ezrin, p-Fak, total Fak, p-Src, and total Src were detected in CAL27 cells transfected with siR-KCNQ1OT1 or siR-control simultaneously with miR-211-5p inhibitors or miR-control inhibitors by Western blot analysis. **d** Correlation analysis between miR-211-5p and KCNQ1OT1 was performed in TSCC tissues. **e** Clones formation capacity was detected in CAL27 cells transfected with siR-KCNQ1OT1 or siR-control simultaneously with miR-211-5p inhibitors or miR-control inhibitors

Our previous results demonstrated that KCNQ1OT1 knockdown and miR-211-5p upregulation inhibited TSCC cell proliferation and chemoresistance, so we proposed the following hypothesis: upregulated miR-211-5p and the consequential inhibition of Fak/Src signaling are the main causes for the TSCC cell growth inhibition and chemo-resistance caused by KCNQ1OT1 knockdown. To validate this hypothesis, we first inhibited miR-211-5p expression and performed cell survival assays in TSCC cells under the cisplatin pressure. As shown in Fig. 6a, the inhibition of miR-211-5p enhanced chemo-resistance in CAL27 cells. Then, we concomitantly decreased the expression of miR-211-5p in TSCC cells with KCNQ1OT1 knocked down. Interestingly, although KCNQ1OT1 knockdown significantly increased TSCC cell death during cisplatin treatment, the simultaneous deletion of miR-211-5p completely reversed cisplatin resistance in TSCC cells, indicating that increased miR-211-5p expression were essential for the KCNQ1OT1 knockdown-induced increase in TSCC cell cisplatin sensitivity. Consistent with these results, miR-211-5p knockdown also completely reversed the KCNQ1OT1 knockdown-induced inhibition of cell viability and colony formation in the TSCC cell lines CAL27 (Fig. 6b, e). Importantly, we also sought to determine whether KCNQ1OT1 regulated Ezrin/Fak/Src signaling and whether this regulation was dependent on miR-211-5p in TSCC cells. As expected, the protein expression levels of EZR and the downstream phosphorylation of Fak and Src were decreased when we knocked down KCNQ1OT1. However, the simultaneous knockdown of miR-211-5p in TSCC cells with KCNQ1OT1 knocked down reversed the repression of EZR and phosphorylation of Fak and Src (Fig. 6c). Moreover, we observed an inverse correlation between the expression levels of KCNQ1OT1 and miR-211-5p (*r* = −0.6270, *P* < 0.001) and between KCNQ1OT1 in TSCC tissue samples (Fig. 6d). In summary, these data strongly support the hypothesis that KCNQ1OT1 promotes TSCC cell proliferation and chemo-resistance via the regulation of miR-211-5p mediated Ezrin/Fak/Src signaling.

### Deletion of KCNQ1OT1 inhibits tumorigenicity and chemo-resistance of TSCC in vivo

**Fig. 7:**
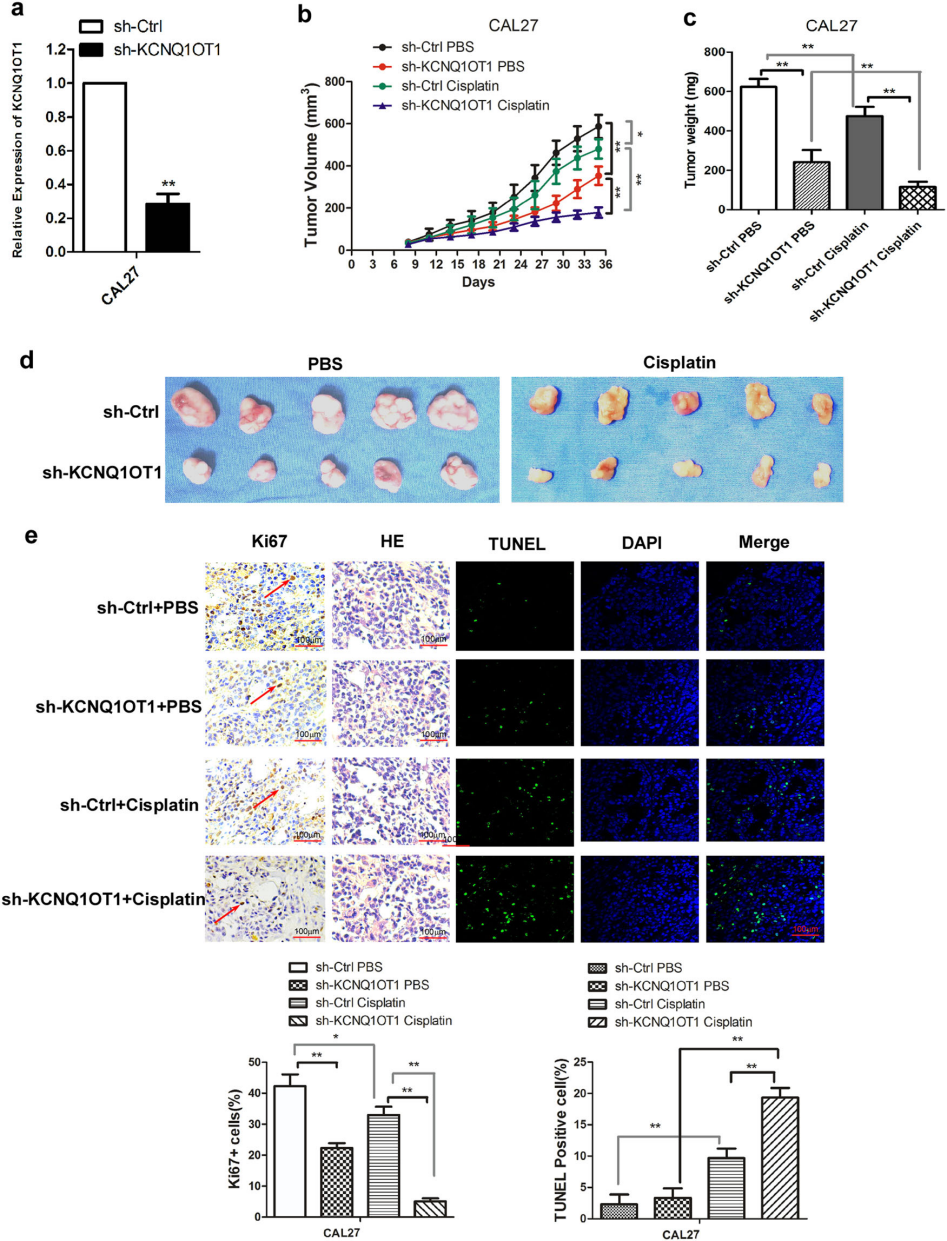
Downregulation of KCNQ1OT1 suppresses tumorigenicity and chemo-resistance of tongue cancer cells in vivo. **a** Construction of KCNQ1OT1 stable knockdown CAL27 cells. **b** The volume of tumors was measured every 3 days. **c** The weight of tumors was measured after the tumors were surgically dissected. **d** Photographs of surgically dissected tumors of each group. **e** The expression of Ki67 in the xenografts was examined by IHC. **f** The apoptosis in the xenografts was detected by TUNEL assay. The histograms showed the score of IHC and proportion of TUNEL-positive cells in each group. The percentage in the histograms indicates the decreased or increased percentage in the cisplatin group compared with the PBS group

To further evaluate the effects of KCNQ1OT1 on TSCC cell tumorigenesis and chemo-resistance in vivo, we generated KCNQ1OT1 stable knockdown CAL27 cells by lentiviral infection (Fig. 7a). CAL27 or control cells with KCNQ1OT1 knocked down were then injected subcutaneously into BALB/c male nude mice. One week after tumor cell inoculation, the nude mice bearing xenografts of CAL27 cells with control shRNA or those with shRNA against KCQ1OT1 (CAL27-sh-KCNQ1OT1) were randomly selected for treatment with either cisplatin or PBS as reported previously^13,20^. Tumor growth in the KCNQ1OT1 knockdown group was substantially suppressed compared with that in the control group (Fig. 7b, d). The tumor weights from the KCNQ1OT1 knockdown group were significantly lower than those of the control group (Fig. 7c). Interestingly, the difference in tumor weight and growth between the KCNQ1OT1 knockdown and control groups was much more significant in the group treated with cisplatin than in the group given PBS (Fig. 7b–d). Moreover, compared with the control group, the KCNQ1OT1 knockdown group tumors exhibited a lower expression of the proliferation marker Ki67 and a higher proportion of cells positive for terminal deoxynucleotidyl transferase (TdT) dUTP nick-end labeling (TUNEL), and these differences were especially significant under paclitaxel treatment (Fig. 7e). These results indicate that KCNQ1OT1 depletion inhibits TSCC cell growth and chemo-resistance in vivo.

## Discussion

The dysregulation of lncRNAs has been reported to be involved in numerous biological processes, such as cell proliferation, cell cycle progression, cell invasion, cell migration, and chemoresistance, in various neoplasms, including breast cancer, gastric cancer, and bladder cancer^21–23^. Therefore, a comprehensive understanding of the regulatory mechanism of lncRNAs may help develop novel, promising therapeutic strategies for the treatment of TSCC. Previous reports have shown that several lncRNAs are involved in the development of TSCC^20,24–26^. For example, Huang found that the NF-KappaB-interacting lncRNA NKILA was downregulated in TSCC and inhibited cell migration and invasion through influencing the epithelial-mesenchymal transition (EMT) that is mediated by the NF-ΚB signaling pathway^25^. Yang demonstrated that the lncRNA UCA1 promoted tongue cancer cell proliferation and metastasis and inhibited their apoptosis through affecting the activation of the WNT/β-catenin signaling pathway^26^. Jia revealed that low expression levels of both miR-26a and MEG3 in TSCC tissues correlated with poor clinical outcome, and miR-26a regulated the methylation of MEG3 by targeting DNA methyltransferase 3B^20^. Studies on the molecular mechanism of lncRNA involvement in TSCC chemo-resistance are limited, so the exploration of comprehensive lncRNA regulatory mechanisms in chemo-resistance is urgently needed.

In this study, we revealed that lncRNA KCNQ1OT1, identified by a lncRNA microarray, was more highly expressed in chemo-insensitive TSCC tissues and two cisplatin-resistant TSCC cells, which were established in our previous study^13,27^. The high expression of KCNQ1OT1 in TSCC contributed to a poor prognosis, indicating KCNQ1OT1 expression as an independent prognostic factor in TSCC patients. KCNQ1OT1 is an antisense transcript to the human KCNQ1 gene and regulates transcription of different target genes through epigenetic modifications. LncRNA KCNQ1OT1 was not only abnormally expressed from the chromosomes in most patients with Beckwith-Wiedemann syndrome, but also played an important role in colorectal carcinogenesis^28,29^. Jin reported that KCNQ1OT1 competed with miR-214 and activated caspase-1 pathway to promote cataractogenesis^30^. The activation of KCNQ1OT1/miR-370/CCNE2 axis resulted in glioma carcinogenesis^31^. Our results indicated that KCNQ1OT1 could promote TSCC cell proliferation and reduce the sensitivity to cisplatin. Ren reported a similar result that KCNQ1OT1 knockdown in lung adenocarcinoma cells suppressed paclitaxel-induced chemo-resistance and contributed to the inhibition of cell proliferation and invasion^32^. These results suggested that lncRNA KCNQ1OT1 functions as an oncogene and plays a critical role in TSCC growth and chemoresistance.

Recent studies have indicated that lncRNAs participate in gene regulation at the pretranscriptional, transcriptional, and post-transcriptional levels, and to a large extent, the level of regulation depends on the cellular location of the lncRNA^33–36^. LncRNAs located in the cytoplasm always function as endogenous miRNA sponges for miRNA response elements (MREs), thereby impairing the function of target mRNA at the post-transcriptional level^35^. In the present study, using FISH assays and RT-qPCR detection of RNAs in the cell cytoplasm or nucleus, we noted that KCNQ1OT1 was a cytoplasmic long noncoding RNA, which suggested that KCNQ1OT1 might exert its function as a competing endogenous RNA (ceRNA) at the post-transcription level. Thus, to verify the ceRNA mechanism of KCNQ1OT1, we first searched for candidate miRNAs.

For ceRNA regulation, MREs are essential for lncRNAs to control the endogenous expression level of miRNAs^36^. A large number of lncRNAs have shown capacity for sponging miRNA to exert functions in tumorigenesis and tumor progression. For example, lncRNA ODRUL can sponge miR-3182 to promote cell proliferation, migration, invasion, and tumor growth^37^. In addition, oxaliplatin resistance was conferred in colon cancer through regulation of the Linc00152-miR-193a-3p-ERBB4-AKT pathway^4^. In addition, Liu reported that the lncRNA SPRY4-IT1 promoted bladder cancer progression by acting as an endogenous miRNA sponge to regulate miR-101-3p, which further affected the expression of its target gene EZH2^38^. Using bioinformatics tools (starBase), dual luciferase reporter and RNA pull-down assays, we confirmed that miR-211-5p directly bound to KCNQ1OT1. In addition, the expression of miR-211-5p was inversely related to that of KCNQ1OT1 in TSCC samples and cell lines. KCNQ1OT1 knockdown combined with miR-211-5p inhibition significantly rescued the reduced cell proliferation and cisplatin resistance induced by KCNQ1OT1 knockdown alone. Our results indicated that KCNQ1OT1 competed with miR-211-5p and exerted its function in TSCC. However, the underlying mechanism by which miR-211-5p suppress TSCC progression is still unclear.

MiRNAs directly bind to the 3′-UTRof downstream targeting genes involved in tumor progression^39^. miR-211-5p overexpression suppressed the proliferation, migration, and invasion of triple-negative breast cancer, renal cancer, and thyroid tumor^40–42^. Moreover, the lncRNA NEAT1/miR-211-5p/HMGA2 axis promoted cell growth and 5-fluorouracil (5-FU) resistance in breast cancer^43^. Nevertheless, reports on the role of miR-211-5p in TSCC are rare. As the bioinformatics analysis and luciferase assay indicated, Ezrin (EZR) was confirmed as a common target gene of miR-211-5p for influencing the malignant behavior of TSCC. EZR is a member of the Ezrin-radixin-moesin (ERM) family, usually thought to be a key cross-linker between membrane proteins and actin filaments, and this gene is highly expressed in a variety of human solid cancers, including TSCC^44^. Previous studies have shown that activated EZR protein plays a key role in chemo-resistance^45^. Our studies revealed that the knockdown of miR-211-5p and rescued the effect of sh-KCNQ1OT1 on TSCC cell growth and chemo-resistance via activation of the Ezrin/Fak/Src signaling pathway. In summary, these data revealed that the lncRNA KCNQ1OT1 effectively sponges miR-211-5p to promote TSCC progression through the Ezrin/Fak/Src signaling pathway.

In conclusion, we identified that KCNQ1OT1 acts as an oncogene, is highly expressed in TSCC tissues, especially in chemo-insensitive TSCC patients, and exerts an important role in the regulation of TSCC chemoresistance. In addition, for the first time, our study suggests a significant role for KCNQ1OT1 in cisplatin resistance via the ceRNA regulatory pathway in TSCC and indicates that this lncRNA competes with miR-211-5p and upregulates Ezrin/Fak/Src signaling to promote TSCC progression. According to our clinical evidence, KCNQ1OT1 acts as an independent prognostic factor for tongue cancer patients, and it may be valuable for predicting chemoresistance.

## Materials and methods

### Microarray analysis

Analyses using Human 8 × 60 K LncRNA expression array provide by KangchengBio Corporation (Shanghai, China) were performed as described previously with the chemo-sensitive sample and the chemo-insensitive sample from the same patient^27^. The cluster map depicting the expression levels of all lncRNAs that were differentially expressed between the chemosensitive sample and the chemo-insensitive sample was generated with DMVS 2.0 software (Chipscreen Biosciences, Shenzhen, China). Biotin labeled total RNA obtained from CAL27 cells transfected with KCNQ1OT1 shRNA or shNC controls was hybridized on an Affymetrix GeneChip® miRNA 3.0 Array. The miRNA array was scanned using the Affymetrix® GeneChip® Scanner 3000 and the results were analyzed by Affymetrix Expression Console.

### Cell culture and drug treatment

The CAL27 and SCC9 cell lines were obtained from ATCC (Manassas, VA, USA). The stable cisplatin-resistant cell lines, CAL27-res and SCC9-res, were established by the selection of CAL27 or SCC9 colonies treated with 10^−7^ to 10^−5^ M cisplatin (Sigma, Carlsbad, CA, USA) as described previously^46^. CAL27 and CAL27-res cells were grown in DMEM medium (Gibco) supplemented with 10% FBS (Invitrogen, Carlsbad, CA, USA). The SCC9 and SCC9-res cells were cultured in DMEM/F-12 (Gibco) supplemented with 10% FBS. Cisplatin was routinely added to the medium every other day and was removed before the experiments were performed.

### Patients and tissue specimens

TSCC specimens (*n* = 102) were collected from Sun Yat-sen Memorial Hospital between 2006 and 2012. Patient outcomes were classified as cisplatin sensitive or insensitive as previously reported^13,27^. The tumor samples were examined by two independent pathologists, and tumor grade was determined according to WHO criteria (2004)^27^. All patients provided informed consent for participation, and approval from the Institutional Research Ethics Committee was obtained.

### Cell transfection

SiRNA targeting KCNQ1OT1 and control siRNAs were obtained from RiboBio (Guangzhou, China). The indicated cells were transfected with 50 nM siRNA using Lipofectamine 3000 (Invitrogen). The targeting sequences were listed as followed: siRNA-1: 5′-GCCAATAGCAACTGACTAA-3′;siRNA-2:5′-GCCACATCTAACACCTATA-3′. miR-211-5p mimics, miR-211-5p inhibitors and miR-controls were purchased from GenePharma (Shanghai, China). MiRNA mimics, miRNA inhibitors and miR-controls were transfected into cells at a concentration of 40 nM using Lipofectamine 3000 (Invitrogen).

### Stable KCNQ1OT1 knockdown cell lines

Plasmids containing specific shRNAs targeting KCNQ1OT1 or an empty vector, pLKO.1, were obtained from GenePharma (Shanghai, China). To establish stable knockdown cell lines, the pLKO.1-shKCNQ1OT1 vector and control shRNA vector plasmid were transfected into 293FT cells using Lipofectamine 2000 (Invitrogen) following the manufacturer’s instructions. Then, CAL27 cells were infected with lentivirus containing the pLKO.1-shKCNQ1OT1 vector and control plasmids and were selected with 2 μg/ml puromycin for 10 days.

### Plasmids transfection

The plasmid vectors pcDNA-dCas9-HA (for CRISPR-based interference) and HUbC-acsa9-VP64 (for CRISPR-based activation) containing each designed sgRNA were purchased from IGEbio (Guangzhou, china). The designed cDNA sequence for each sgRNA was listed in S3-table. The plasmids expressed Cas9-VP64 fusion protein (for CRISPR-based activation) or dCas9 protein (for CRISPR-based interference) were transiently transfected in tongue cancer cells with Lipofectamine2000 (Invitrogen, Carlsbad, CA, USA) according to the manufacturer’ sinstructions.

### Nuclear fraction

The cellular fractionation was performed with a PARIS Kit according to the instructions of the manufacturer (Ambion, Austin, TX). Cells (1 × 10^7^) were collected, resuspended in 1 ml of ice-cold cell fractionation buffer and incubated for 15 min on ice. Then, the cells were centrifuged for 15 min at 500 *g*; the supernatant and nuclear pellet were reserved for RNA extraction using TRIzol LS and TRIzol reagent (Invitrogen, USA) using a previously established protocol^47^.

### Real-time quantitative PCR

Total RNA was isolated from cells or tissues using TRIzol reagent (Invitrogen, USA) and then was converted to cDNA using an M-MLV Reverse Transcriptase Kit (Invitrogen, USA). Real-time PCR analyses were carried out in triplicate for each sample using SYBR Green PCR Master Mix (TOYOBO) on a LightCycler 480 system (Roche). All primers are listed in S4-table, and glyceraldehyde-3-phosphate dehydrogenase (GAPDH) served as the endogenous control. For detecting miRNA expression level, cDNA was synthesized using a TaqMan® miRNA reverse transcription kit (Applied Biosystems, Foster City, CA, USA), and U6 small nuclear RNA served as the endogenous control.

### Western blot analysis

Cells were lysed in chilled RIPA buffer (Beyotime) supplemented with 1 mmol/L protease inhibitor mixture (Sigma-Aldrich). An equal amount of each protein sample was separated on a 10% SDS-PAGE gel and was transferred onto a PVDF membrane (Millipore Corporation, USA). The membranes were blocked with 5% nonfat dry milk at RT for 1 h and were incubated with specific primary antibodies overnight (S5-table), followed by an incubation with HRP-conjugated anti-mouse or anti-rabbit secondary antibodies (Proteintech, USA). The peroxidase reaction was detected using enhanced chemiluminescence reagent (ECL, Thermo, Rockford, USA).

### Cell proliferation assay

Cell proliferation was measured by performing the MTS assay and 5-ethynyl-2′-deoxyuridine (EdU) incorporation assay, using an MTS assay kit (Promega, Tokyo, Japan) and EdU assay kit (Life Technologies Corporation, USA), respectively.

For the MTS assay, 1000 cells were seeded into a 96-well plate and were cultured for 6 days at 37 °C. Then, 20 µl of MTS solution was added to each well, followed by a 1 h incubation at 37 °C, and the absorbance at 490 nm was measured.

For EdU incorporation assays, cells were cultured in 24-well plates, and 10 μM EdU was added to each well. Then, the cells were cultured for 2 h at 37 °C and were fixed with 4% formaldehyde for 20 min at RT. After washing with PBS, the incorporated EdU was detected with a Click-iT® EdU kit for 30 min at RT, and subsequently, the cells were stained with Hoechst 33342 for 20 min and were visualized using a fluorescence microscope (Olympus, Tokyo, Japan). The EdU incorporation rate was calculated as the ratio of the number of EdU-positive cells (red cells) to the total number of Hoechst 33342-positive cells (blue cells).

To assay clonogenicity, 500 cells were seeded in each well of a 6-well plate, and then, after 7 days of culture, the cells were fixed and stained with 0.5% crystal violet solution. Colonies with a diameter >50 µm were counted.

### Cell survival assays and apoptosis analysis

Briefly, cells were cultured in a 96-well plate overnight at a concentration of 2000 cells/ml per well and were treated with the indicated concentrations of cisplatin (2, 4, 6, 8, and 10 µM) for 24 h. Then, 20 µl of MTS solution was added to each well, followed by a 1 h incubation at 37 °C, and the absorbance at 490 nm was measured.

Cell apoptosis assays were performed using an Annexin V-FITC/PI kit according to the manufacturer’s instructions (KeyGEN BioTECH, China). Harvested cells were analyzed with flow cytometry (FACScan, BD Biosciences, USA).

TUNEL assays were conducted by using the In Situ Cell Death Detection kit (Roche) according to the manufacturer’s instructions.

### Luciferase assay

We cloned wild-type KCNQ1OT1 with potential miR-211-5p and miR-204 binding sites or mutants of each site into pMIR-REPORT plasmids (Promega, Madison, WI, USA). Similarly, the predicted miR-211-5p and miR-204 response elements (wild-type or mutant) in the 3′-UTR of Ezrin(EZR) were amplified and were cloned into a pMIR-REPORT vector. HEK293T cells were placed in a 24-well plate and were co-transfected with luciferase plasmids and miR-211-5p, control miRNA. After 48 h of transfection, firefly and Renilla luciferase activities were detected with a dual luciferase reporter assay system (Promega).

### RNA-binding protein immunoprecipitation (RIP) assay

For anti-AGO2 RIP, CAL27, and SCC9 cells were transfected with hsa-miR-197-3p, hsa-miR-761, hsa-miR-204-5p, hsa-miR-211-5p, hsa-miR-134-5p, or microRNA negative control. After 48 h transfection, cells were performed for RIP assays using a Magna RIP RNA-Binding Protein Immunoprecipitation kit as previously reported (Millipore, Billerica, MA, USA)^47^. Briefly, Ago2 antibody (Cell Signaling Technology, Beverly, MA) and normal rabbit IgG (Proteintech), as the negative control, were conjugated to magnetic beads and were incubated with the cell extract in RIP buffer. The immunoprecipitated RNAs were isolated and were examined by real-time PCR. The input controls and IgG controls were assayed simultaneously to demonstrate that the detected signals were the result of RNAs specifically binding to Ago2.

### IHC staining and scoring analyses

The scores from immunohistochemistry experiments were calculated as described previously^48^. We used anti-Ki67 antibodies (ready to use; Zhongshan Bio-Tech) to detect the expression of Ki67 in the mouse xenografts. The sections were visualized using a NikonECLIPSE Ti microscope system and were processed with Nikon software.

### In situ hybridization (ISH)

An ISH assay was performed according to the manufacturer’s protocol (Exiqon, Vedbaek, Denmark). Briefly, after dewaxing TSCC specimens were hybridized to KCNQ1OT1 using a 5′digoxin-labeled LNATM-modified KCNQ1OT1 probe (Exiqon, Vedbaek, Danmark). Then, the digoxigenin was recognized by a specific anti-DIG antibody to which alkaline phosphatase was conjugated. Cell nuclei were counterstained with hematoxylin.High expression, positive cells ≥30%; lowexpression, positive cells <30%.

### Mice xenograft assay

All animal procedures were approved by the Institutional Animal Care and Use Committee of Sun Yat-sen University. Four-week-old BALB/c nude mice were purchased from the Experimental Animal Center of Sun Yat-sen University and were housed in specific pathogen-free (SPF) barrier facilities. CAL27 cells (5 × 10^6^) infected with shRNA-KCNQ1OT1 or empty vector were subcutaneously injected into nude mice. Beginning 8 days after inoculation and continuing until day 32, cisplatin (5 mg/kg) was injected intraperitoneally every 3 days into nude mice bearing CAL27-sh-control or CAL27-sh-KCNQ1OT1 xenografts. Tumor volume was measured every 3 days and was calculated by the following formula: volume (mm^3^) = (length × width^2^)/2. Thirty-five days after implantation, the xenografts were dissected carefully and were fixed in 4% paraformaldehyde.

### Statistical analysis

Statistical analysis was performed using SPSS 20.0 software (SPSS Inc., Chicago, IL). All data are expressed as the group mean ± standard deviation (SD). The χ2 test was used to analyze relationships between related proteins. Kaplan-Meier survival curves were plotted, and the log-rank test was used. All experiments were performed at least three times. The results of the experiments are expressed as the means ± SD. *P* < 0.05 was considered statistically significant.

## Electronic supplementary material

supplementary figure legends

Supplementary Figure 1

Supplementary Figure 2

Supplementary Figure 3

Supplementary Figure 4

Supplementary Figure 5

S2 table

S5 table

S3 table

S4 table
