# Supplementary material for: LncRNA KCNQ1OT1 regulates proliferation and cisplatin resistance in tongue cancer via miR-211-5p mediated Ezrin/Fak/Src signaling
Source: Cell Death Dis. 2018 Jul 3;9(7):742. doi: 10.1038/s41419-018-0793-5 (PMC6030066; doi:10.1038/s41419-018-0793-5)
Supplement: Supplementary file 1 — supplementary figure legends [file 41419_2018_793_MOESM1_ESM.docx]

**Supplementary Figure 1** (**a**) Number of clones was counted in CAL27 and SCC9 cells transfected with KCNQ1OT1 siRNAs or control siRNA. (**b**) Edu positive cells were counted in CAL27 and SCC9 cells transfected with KCNQ1OT1 siRNAs or control siRNA (c and d). Flow cytometer was used for analyzing the percentage of apoptotic cells in CAL27 (**c**) and SCC9 (**d**) cells transfected with control or KCNQ1OT1 siRNAs were treated with 4μM cisplatin(for CAL27 cells) or 8 μM cisplatin(for SCC9 cells) for 24 hrs.

**Supplementary Figure 2** KCNQ1OT1 downregulation inhibited TSCC growth and chemo-resistance

1. Inhibition of KCNQ1OT1expression using the designed sgRNAs in CAL27-res and SCC9-res cells was verified by RT-qPCR. (**b**) Effect of KCNQ1OT1 downregulation on cell proliferation of CAL27 and SCC9 cells was measured by the MTS assay. (**c**) Effect of KCNQ1OT1 downregulation on colony formation was measured in CAL27-res and SCC9-res cells. (**d**) The cell viability of CAL27-res and SCC9-res cells transfected with plamids containing sgRNAs were examined by EdU assay. (**e**) Effect of KCNQ1OT1 downregulation on cell viability under cisplatin pressure was measured by the MTS assay. (**f**) The CAL27-res and SCC9-res cells transfected with plamids containing sgRNAs were treated with 4μM cisplatin(for CAL27-res cells) or 8 μM cisplatin(for SCC9-res cells) for 24 hrs. The percentage of apoptotic cells was analyzed by flow cytometer.

**Supplementary Figure 3** KCNQ1OT1 upregualtion promotes tongue cancer cell proliferation and enhance the sensitivity to cispaltin

(**a**) Activation of KCNQ1OT1expression using the designed sgRNAs in CAL27 and SCC9 cells was verified by RT-qPCR. (**b**) Effect of KCNQ1OT1 upregulation on cell proliferation of CAL27 and SCC9 cells was measured by the MTS assay. (**c**) Effect of KCNQ1OT1 upregulation on colony formation was measured in SCC9 and CAL27 cells. (**d**) The cell viability of CAL27 and SCC9 cells transfected with plamids containing sgRNAs were examined by EdU assay. (**e**) Effect of KCNQ1OT1 upregulation on cell viability under cisplatin pressure was measured by the MTS assay. (**f**) The CAL27 and SCC9 cells transfected with plamids containing sgRNAs were treated with 4μM cisplatin(for CAL27 cells) or 8 μM cisplatin(for SCC9 cells) for 24 hrs. The percentage of apoptotic cells was analyzed by flow cytometer.

**Supplementary Figure 4** (**a**) miRNA microarrays were used to screen differentially expressed miRNAs associated with KCNQ1OT1 in the paired sh-KCNQ1OT1 and sh-NC CAL27 cells. (**b**) The top ten upregulated and downregulated miRNAs were validated in CAL27 cells transfected with KCNQ1OT1 shRNAs or control shRNA.

**Supplementary Figure 5** miR-211-5p suppress SCC9 proliferation and chemo-resistance by targeting Ezrin/Fak/Src signaling (**a**) Cell viability was detected by MTS assays in SCC9 cells transfected with or without miR-211-5p inhibitors. (**b**) Cell viability was detected by MTS assays in SCC9-res cells transfected with or without miR-211-5p mimics. (**c**) Cell survival rate was examined by MTS assays under cisplatin pressure in SCC9 cells transfected with or without miR-211-5p inhibitors. (**d**) Cell survival rate was examined by MTS assays under cisplatin pressure in SCC9-res cells transfected with or without miR-211-5p mimics. (**e**) The expression of Ezrin, p-Fak, total Fak, p-Src and total Src were detected in miR-211-5p-depleted SCC9 cells and miR-211-5p overexpressed SCC9-res cells by western blot. (**f**) Clones formation was performed in SCC9 cells transfected with miR-211-5p inhibitors.
