# Supplementary figures and images for: LncRNA KCNQ1OT1 regulates proliferation and cisplatin resistance in tongue cancer via miR-211-5p mediated Ezrin/Fak/Src signaling
Source: Cell Death Dis. 2018 Jul 3;9(7):742. doi: 10.1038/s41419-018-0793-5 (PMC6030066; doi:10.1038/s41419-018-0793-5)

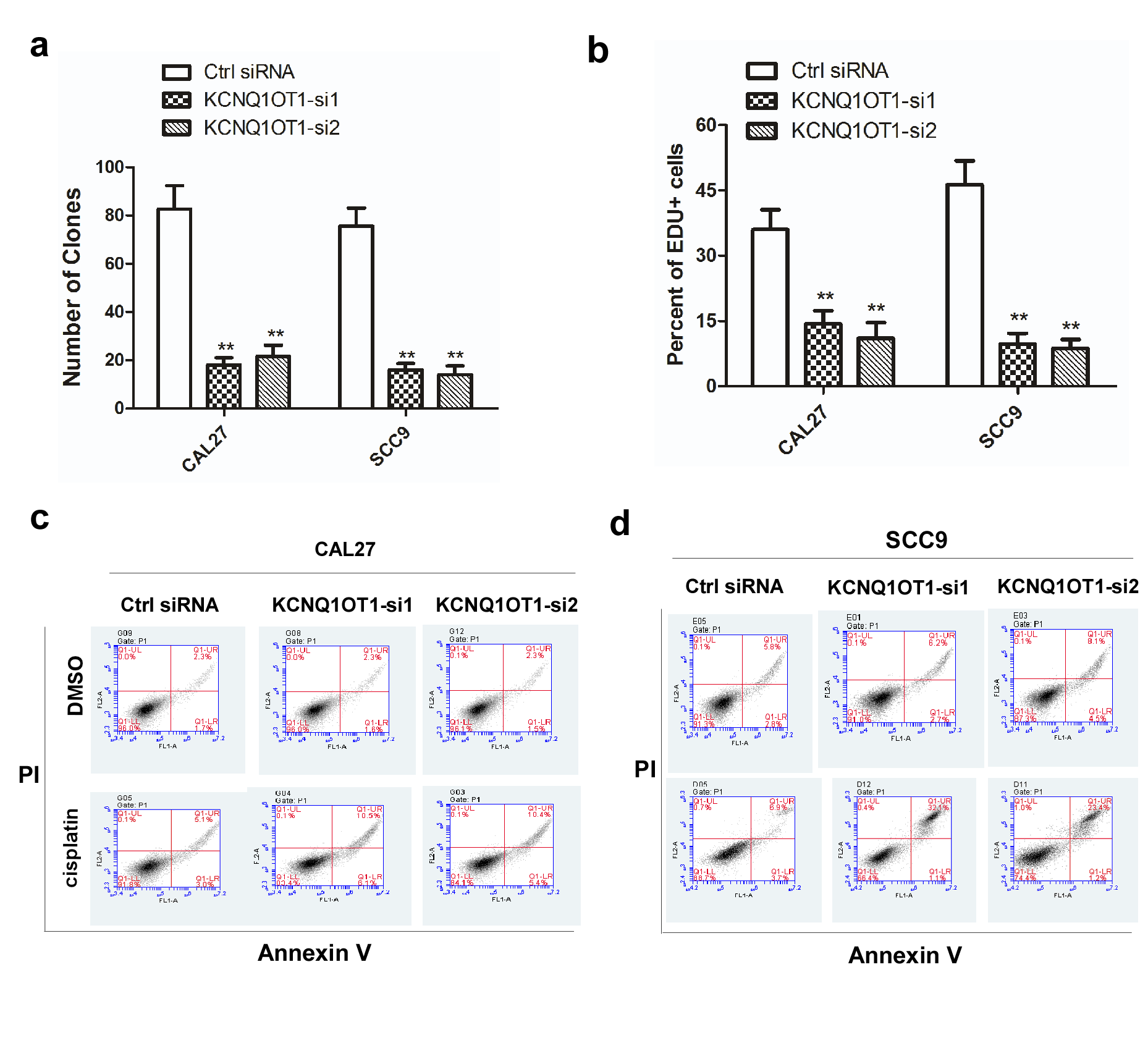

Supplement: Supplementary file 2 — Supplementary Figure 1 [file 41419_2018_793_MOESM2_ESM.tif]

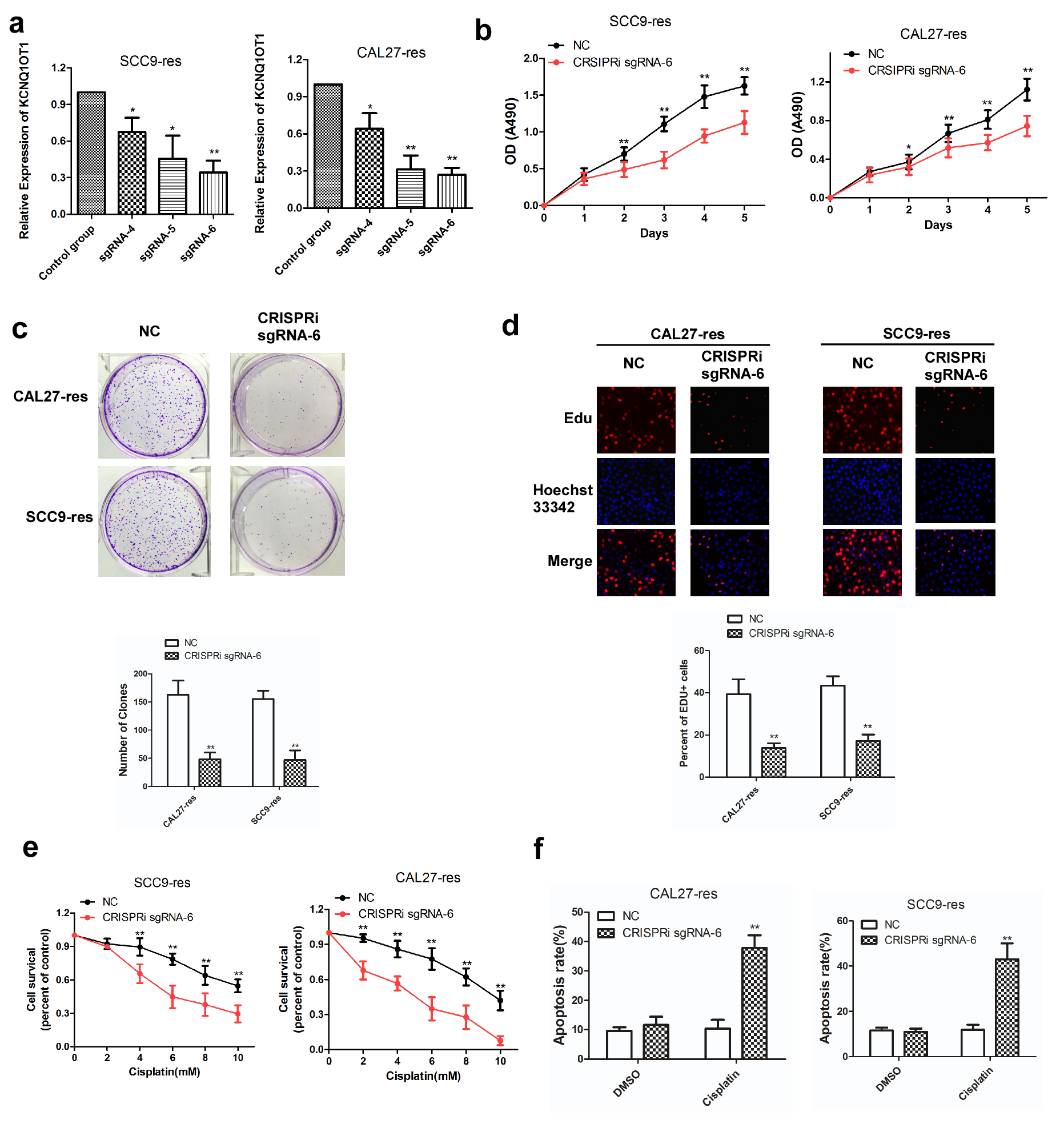

Supplement: Supplementary file 3 — Supplementary Figure 2 [file 41419_2018_793_MOESM3_ESM.tif]

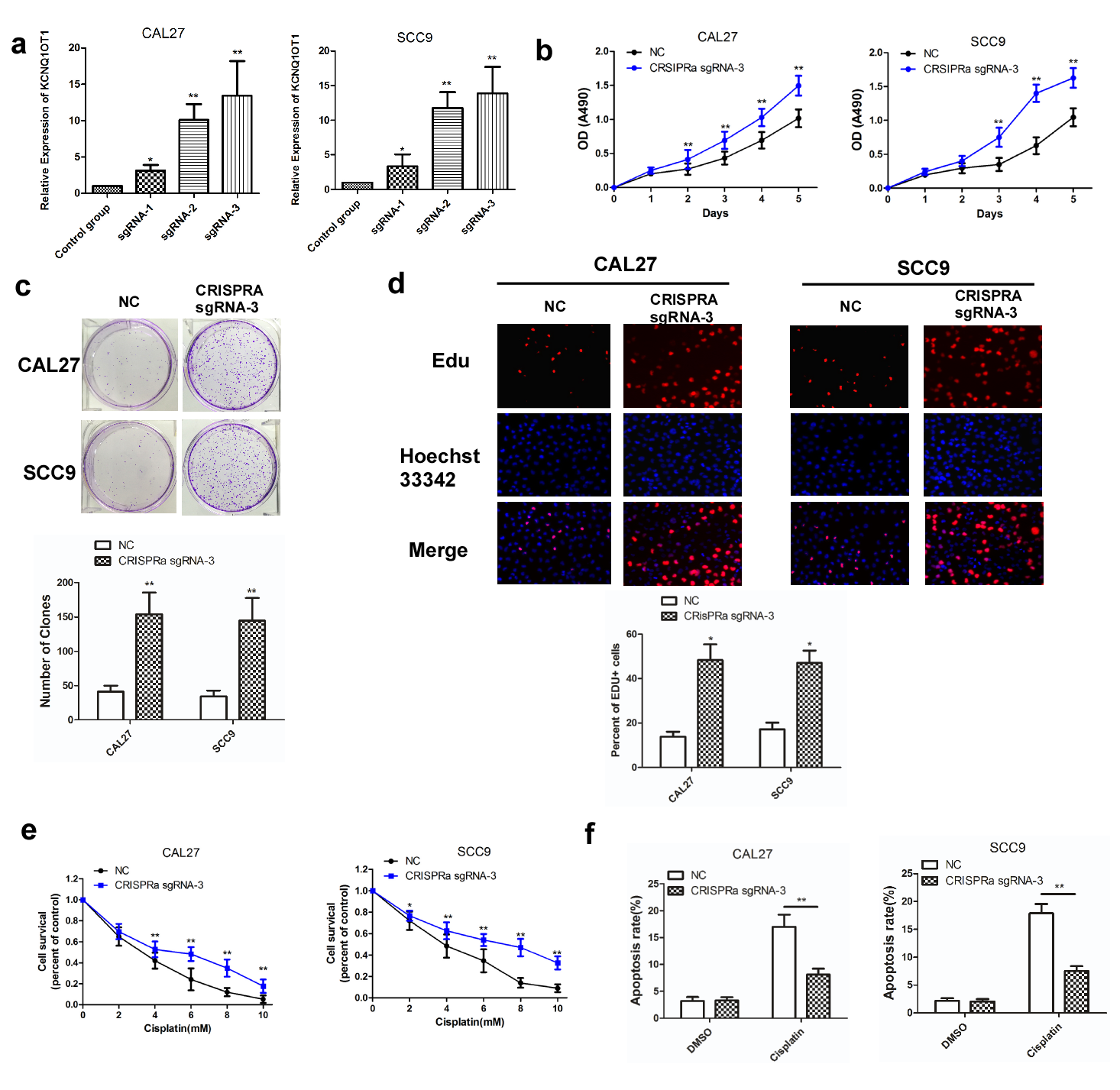

Supplement: Supplementary file 4 — Supplementary Figure 3 [file 41419_2018_793_MOESM4_ESM.tif]

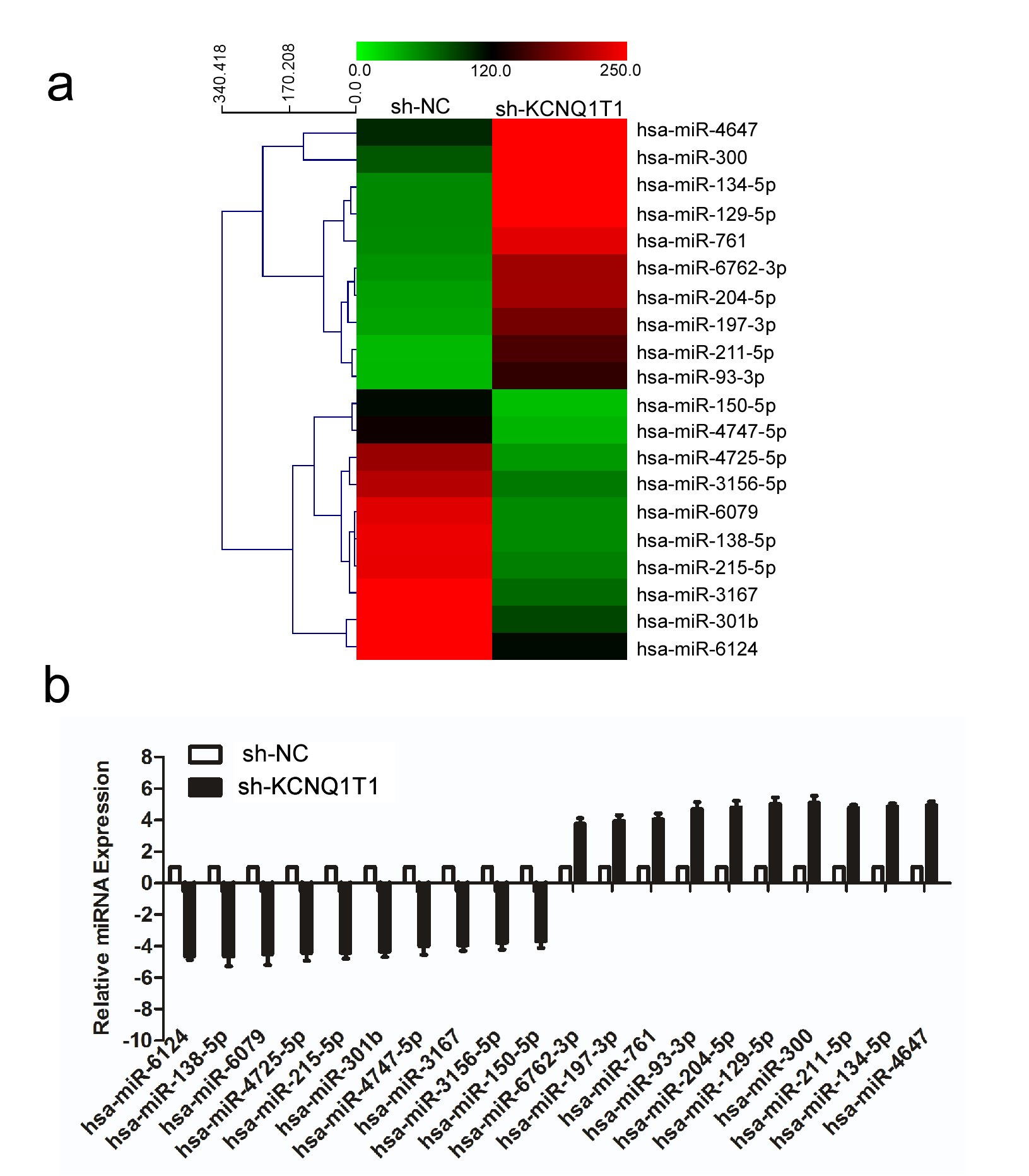

Supplement: Supplementary file 5 — Supplementary Figure 4 [file 41419_2018_793_MOESM5_ESM.tif]

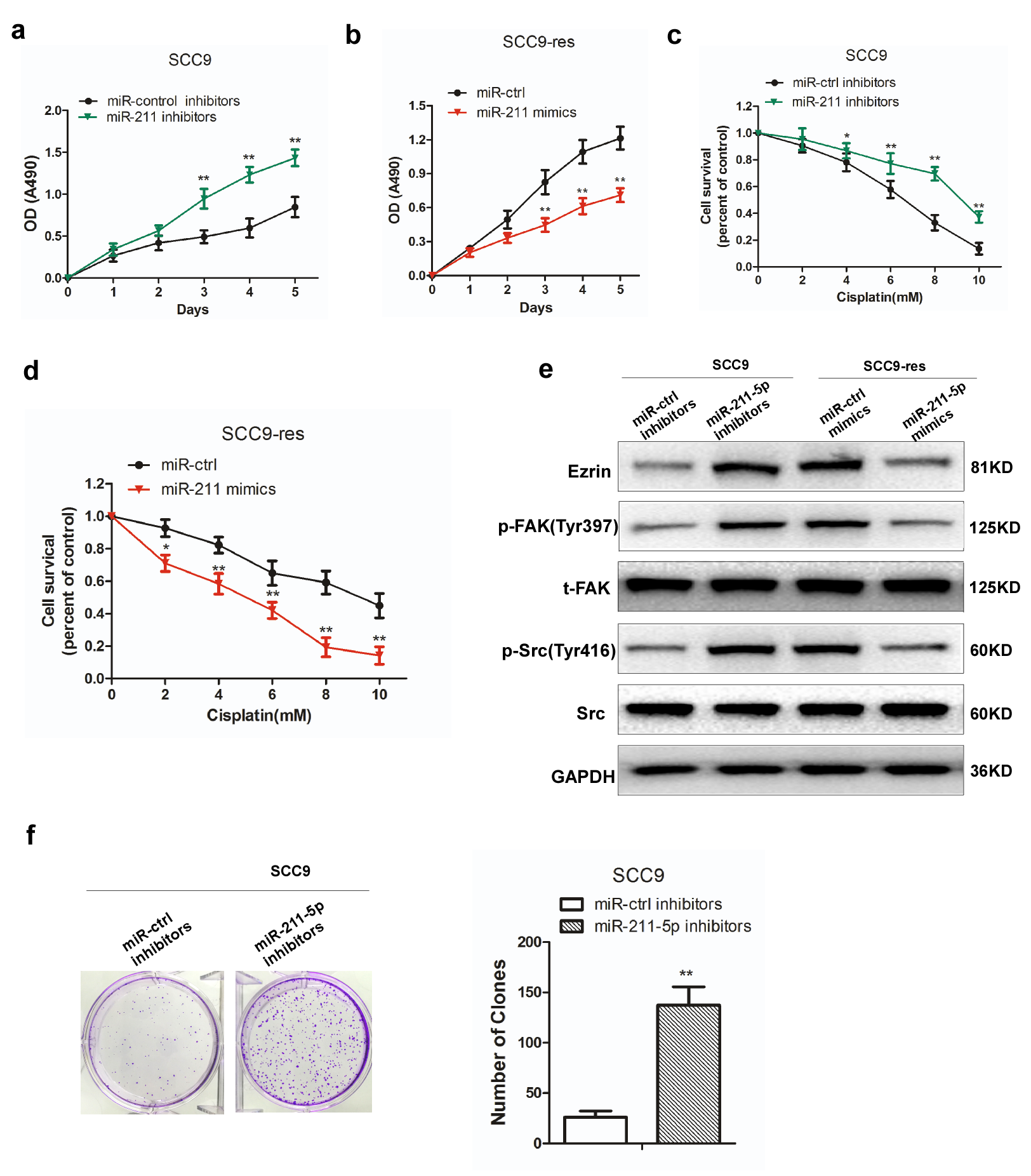

Supplement: Supplementary file 6 — Supplementary Figure 5 [file 41419_2018_793_MOESM6_ESM.tif]
