# Supplementary material for: LncRNA KCNQ1OT1 regulates proliferation and cisplatin resistance in tongue cancer via miR-211-5p mediated Ezrin/Fak/Src signaling
Source: Cell Death Dis. 2018 Jul 3;9(7):742. doi: 10.1038/s41419-018-0793-5 (PMC6030066; doi:10.1038/s41419-018-0793-5)
Supplement: Supplementary file 7 — S2 table [file 41419_2018_793_MOESM7_ESM.doc]

| name | mirAccession | geneName | targetSites | bioComplex | clipReadNum | cancerNum |
| --- | --- | --- | --- | --- | --- | --- |
| hsa-miR-34a-5p | MIMAT0000255 | KCNQ1OT1 | 1 | 2 | 0 | 2 |
| hsa-miR-761 | MIMAT0010364 | KCNQ1OT1 | 2 | 2 | 0 | 0 |
| hsa-miR-197-3p | MIMAT0000227 | KCNQ1OT1 | 1 | 2 | 0 | 2 |
| hsa-miR-3118 | MIMAT0014980 | KCNQ1OT1 | 1 | 2 | 0 | -1 |
| hsa-miR-214-3p | MIMAT0000271 | KCNQ1OT1 | 2 | 2 | 0 | 0 |
| hsa-miR-4735-3p | MIMAT0019861 | KCNQ1OT1 | 1 | 1 | 10 | -1 |
| hsa-miR-29c-3p | MIMAT0000681 | KCNQ1OT1 | 2 | 2 | 0 | 1 |
| hsa-miR-29b-3p | MIMAT0000100 | KCNQ1OT1 | 2 | 2 | 0 | 1 |
| hsa-miR-346 | MIMAT0000773 | KCNQ1OT1 | 1 | 2 | 0 | 0 |
| hsa-miR-107 | MIMAT0000104 | KCNQ1OT1 | 1 | 2 | 0 | 2 |
| hsa-miR-146b-5p | MIMAT0002809 | KCNQ1OT1 | 1 | 2 | 0 | 2 |
| hsa-miR-4295 | MIMAT0016844 | KCNQ1OT1 | 1 | 2 | 0 | 0 |
| hsa-miR-130a-3p | MIMAT0000425 | KCNQ1OT1 | 1 | 2 | 0 | 0 |
| hsa-miR-326 | MIMAT0000756 | KCNQ1OT1 | 4 | 2 | 0 | 1 |
| hsa-miR-34c-5p | MIMAT0000686 | KCNQ1OT1 | 1 | 2 | 0 | 0 |
| hsa-miR-125b-5p | MIMAT0000423 | KCNQ1OT1 | 1 | 2 | 0 | 1 |
| hsa-let-7a-5p | MIMAT0000062 | KCNQ1OT1 | 1 | 4 | 0 | 4 |
| hsa-miR-3167 | MIMAT0015042 | KCNQ1OT1 | 1 | 2 | 0 | 0 |
| hsa-miR-148b-3p | MIMAT0000759 | KCNQ1OT1 | 2 | 2 | 0 | 3 |
| hsa-let-7i-5p | MIMAT0000415 | KCNQ1OT1 | 1 | 4 | 0 | 2 |
| hsa-miR-4500 | MIMAT0019036 | KCNQ1OT1 | 1 | 4 | 0 | -1 |
| hsa-miR-18a-5p | MIMAT0000072 | KCNQ1OT1 | 1 | 1 | 10 | 2 |
| hsa-miR-19a-3p | MIMAT0000073 | KCNQ1OT1 | 1 | 2 | 0 | 2 |
| hsa-miR-19b-3p | MIMAT0000074 | KCNQ1OT1 | 1 | 2 | 0 | 2 |
| hsa-miR-370-3p | MIMAT0000722 | KCNQ1OT1 | 3 | 4 | 0 | 0 |
| hsa-miR-329-3p | MIMAT0001629 | KCNQ1OT1 | 1 | 2 | 0 | 0 |
| hsa-miR-376c-3p | MIMAT0000720 | KCNQ1OT1 | 1 | 2 | 0 | 0 |
| hsa-miR-134-5p | MIMAT0000447 | KCNQ1OT1 | 1 | 2 | 0 | 0 |
| hsa-miR-377-3p | MIMAT0000730 | KCNQ1OT1 | 2 | 4 | 0 | 0 |
| hsa-miR-211-5p | MIMAT0000268 | KCNQ1OT1 | 1 | 2 | 0 | 3 |
| hsa-miR-7-5p | MIMAT0000252 | KCNQ1OT1 | 2 | 2 | 0 | 1 |
| hsa-miR-138-5p | MIMAT0000430 | KCNQ1OT1 | 1 | 2 | 0 | 1 |
| hsa-miR-328-3p | MIMAT0000752 | KCNQ1OT1 | 2 | 4 | 0 | 1 |
| hsa-miR-140-5p | MIMAT0000431 | KCNQ1OT1 | 2 | 4 | 0 | 2 |
| hsa-miR-4725-5p | MIMAT0019843 | KCNQ1OT1 | 3 | 5 | 10 | -1 |
| hsa-miR-152-3p | MIMAT0000438 | KCNQ1OT1 | 2 | 2 | 0 | 2 |
| hsa-miR-454-3p | MIMAT0003885 | KCNQ1OT1 | 1 | 2 | 0 | 2 |
| hsa-miR-301a-3p | MIMAT0000688 | KCNQ1OT1 | 1 | 2 | 0 | 2 |
| hsa-miR-338-3p | MIMAT0000763 | KCNQ1OT1 | 2 | 2 | 0 | 1 |
| hsa-miR-187-3p | MIMAT0000262 | KCNQ1OT1 | 1 | 2 | 0 | 3 |
| hsa-miR-4319 | MIMAT0016870 | KCNQ1OT1 | 1 | 2 | 0 | -1 |
| hsa-miR-24-3p | MIMAT0000080 | KCNQ1OT1 | 2 | 4 | 0 | 2 |
| hsa-miR-27a-3p | MIMAT0000084 | KCNQ1OT1 | 1 | 2 | 0 | 2 |
| hsa-miR-330-5p | MIMAT0004693 | KCNQ1OT1 | 4 | 2 | 0 | 4 |
| hsa-let-7e-5p | MIMAT0000066 | KCNQ1OT1 | 1 | 4 | 0 | 4 |
| hsa-miR-125a-5p | MIMAT0000443 | KCNQ1OT1 | 1 | 2 | 0 | 0 |
| hsa-miR-216b-5p | MIMAT0004959 | KCNQ1OT1 | 1 | 2 | 0 | 0 |
| hsa-miR-128-3p | MIMAT0000424 | KCNQ1OT1 | 1 | 2 | 0 | 2 |
| hsa-miR-153-3p | MIMAT0000439 | KCNQ1OT1 | 1 | 2 | 0 | 1 |
| hsa-miR-149-5p | MIMAT0000450 | KCNQ1OT1 | 1 | 2 | 0 | 1 |
| hsa-miR-103a-3p | MIMAT0000101 | KCNQ1OT1 | 1 | 2 | 0 | 3 |
| hsa-miR-499a-5p | MIMAT0002870 | KCNQ1OT1 | 1 | 2 | 0 | 0 |
| hsa-miR-124-3p | MIMAT0000422 | KCNQ1OT1 | 1 | 4 | 0 | 0 |
| hsa-let-7c-5p | MIMAT0000064 | KCNQ1OT1 | 1 | 4 | 0 | 4 |
| hsa-miR-301b | MIMAT0004958 | KCNQ1OT1 | 1 | 2 | 0 | 2 |
| hsa-miR-130b-3p | MIMAT0000691 | KCNQ1OT1 | 1 | 2 | 0 | 2 |
| hsa-miR-3619-5p | MIMAT0017999 | KCNQ1OT1 | 2 | 2 | 0 | 1 |
| hsa-let-7b-5p | MIMAT0000063 | KCNQ1OT1 | 1 | 4 | 0 | 3 |
| hsa-miR-425-5p | MIMAT0003393 | KCNQ1OT1 | 1 | 2 | 0 | 2 |
| hsa-let-7g-5p | MIMAT0000414 | KCNQ1OT1 | 1 | 4 | 0 | 3 |
| hsa-miR-4458 | MIMAT0018980 | KCNQ1OT1 | 1 | 4 | 0 | -1 |
| hsa-miR-449a | MIMAT0001541 | KCNQ1OT1 | 1 | 2 | 0 | 0 |
| hsa-miR-449b-5p | MIMAT0003327 | KCNQ1OT1 | 1 | 2 | 0 | 0 |
| hsa-miR-874-3p | MIMAT0004911 | KCNQ1OT1 | 1 | 2 | 0 | 1 |
| hsa-miR-145-5p | MIMAT0000437 | KCNQ1OT1 | 2 | 4 | 0 | 0 |
| hsa-miR-146a-5p | MIMAT0000449 | KCNQ1OT1 | 1 | 2 | 0 | 4 |
| hsa-miR-339-5p | MIMAT0000764 | KCNQ1OT1 | 1 | 2 | 0 | 1 |
| hsa-miR-148a-3p | MIMAT0000243 | KCNQ1OT1 | 2 | 2 | 0 | 1 |
| hsa-miR-3666 | MIMAT0018088 | KCNQ1OT1 | 1 | 2 | 0 | 0 |
| hsa-miR-183-5p | MIMAT0000261 | KCNQ1OT1 | 2 | 4 | 0 | 2 |
| hsa-miR-335-5p | MIMAT0000765 | KCNQ1OT1 | 3 | 4 | 0 | 3 |
| hsa-miR-29a-3p | MIMAT0000086 | KCNQ1OT1 | 2 | 2 | 0 | 3 |
| hsa-miR-490-3p | MIMAT0002806 | KCNQ1OT1 | 1 | 4 | 0 | 1 |
| hsa-miR-383-5p | MIMAT0000738 | KCNQ1OT1 | 1 | 2 | 0 | 0 |
| hsa-miR-486-5p | MIMAT0002177 | KCNQ1OT1 | 1 | 2 | 0 | 2 |
| hsa-miR-491-5p | MIMAT0002807 | KCNQ1OT1 | 1 | 2 | 0 | 1 |
| hsa-miR-876-5p | MIMAT0004924 | KCNQ1OT1 | 1 | 2 | 0 | 0 |
| hsa-miR-204-5p | MIMAT0000265 | KCNQ1OT1 | 1 | 2 | 0 | 1 |
| hsa-let-7f-5p | MIMAT0000067 | KCNQ1OT1 | 1 | 4 | 0 | 4 |
| hsa-let-7d-5p | MIMAT0000065 | KCNQ1OT1 | 1 | 4 | 0 | 2b |
| hsa-miR-27b-3p | MIMAT0000419 | KCNQ1OT1 | 1 | 2 | 0 | 0 |
| hsa-miR-455-5p | MIMAT0003150 | KCNQ1OT1 | 1 | 2 | 0 | 1 |
| hsa-miR-362-3p | MIMAT0004683 | KCNQ1OT1 | 1 | 2 | 0 | 3 |
| hsa-miR-98-5p | MIMAT0000096 | KCNQ1OT1 | 1 | 4 | 0 | 3 |
| hsa-miR-421 | MIMAT0003339 | KCNQ1OT1 | 1 | 2 | 0 | 4 |
| hsa-miR-384 | MIMAT0001075 | KCNQ1OT1 | 1 | 2 | 0 | 0 |
| hsa-miR-18b-5p | MIMAT0001412 | KCNQ1OT1 | 1 | 1 | 10 | 0 |
| hsa-miR-504-5p | MIMAT0002875 | KCNQ1OT1 | 3 | 5 | 10 | 0 |
| hsa-miR-506-3p | MIMAT0002878 | KCNQ1OT1 | 1 | 4 | 0 | 0 |
