# Supplementary material for: LncRNA KCNQ1OT1 regulates proliferation and cisplatin resistance in tongue cancer via miR-211-5p mediated Ezrin/Fak/Src signaling
Source: Cell Death Dis. 2018 Jul 3;9(7):742. doi: 10.1038/s41419-018-0793-5 (PMC6030066; doi:10.1038/s41419-018-0793-5)
Supplement: Supplementary file 8 — S5 table [file 41419_2018_793_MOESM8_ESM.doc]

| Primary antibodies used in the study. | | | |
| --- | --- | --- | --- |
| Antibody | MV | Sources (Catalogue #) | Dilution |
|  | (KDa) |  |  |
| cleaved PARP | 89 | Cell Signaling Technology (#5625) (Beverly, MA) | WB1:1000 |
| cleaved caspase-9 | 37 | Cell Signaling Technology (#7237) (Beverly, MA) | WB1:1000 |
| cleaved caspase-7 | 20 | Cell Signaling Technology (#9194) (Beverly, MA) | WB1:1000 |
| cleaved caspase-3 | 20 | Cell Signaling Technology (#9661) (Beverly, MA) | WB1:1000 |
| Ezrin | 81 | Cell Signaling Technology (#3145) (Beverly, MA) | WB1:1000 |
| p-Fak (Tyr397) | 125 | Cell Signaling Technology (#8556) (Beverly, MA) | WB:1:1000 |
| Fak | 125 | Cell Signaling Technology (#3285) (Beverly, MA) | WB:1:2000 |
| p-Src(Tyr416) | 60 | Cell Signaling Technology (#6943) (Beverly, MA) | WB:1:1000 |
| Src | 60 | Cell Signaling Technology (#2108) (Beverly, MA) | WB:1:1000 |
| Ki67 |  | Zhongshan Bio-Tech(Beijing,China) | IHC: 1:800 |
| GAPDH | 36 | Proteintech,( #10494) (Chicago, IL) | WB:1:5000 |
